# Supplementary figures and images for: Assessing the value of complex refractive index and particle density for calibration of low-cost particle matter sensor for size-resolved particle count and PM2.5 measurements
Source: PLoS One. 2021 Nov 11;16(11):e0259745. doi: 10.1371/journal.pone.0259745 (PMC8584671; doi:10.1371/journal.pone.0259745)

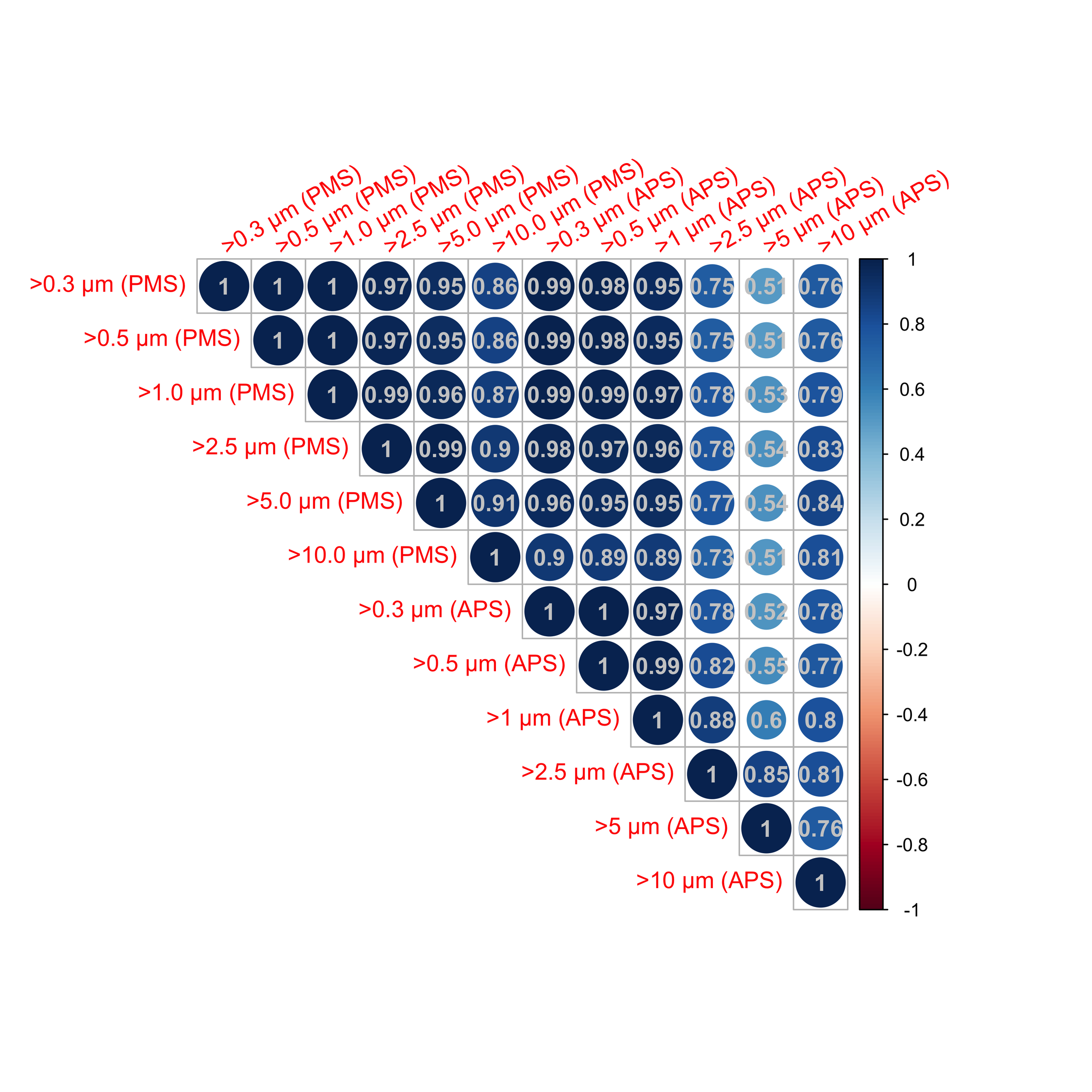

Supplement: S1 Fig — (TIF) [file pone.0259745.s001.tif]

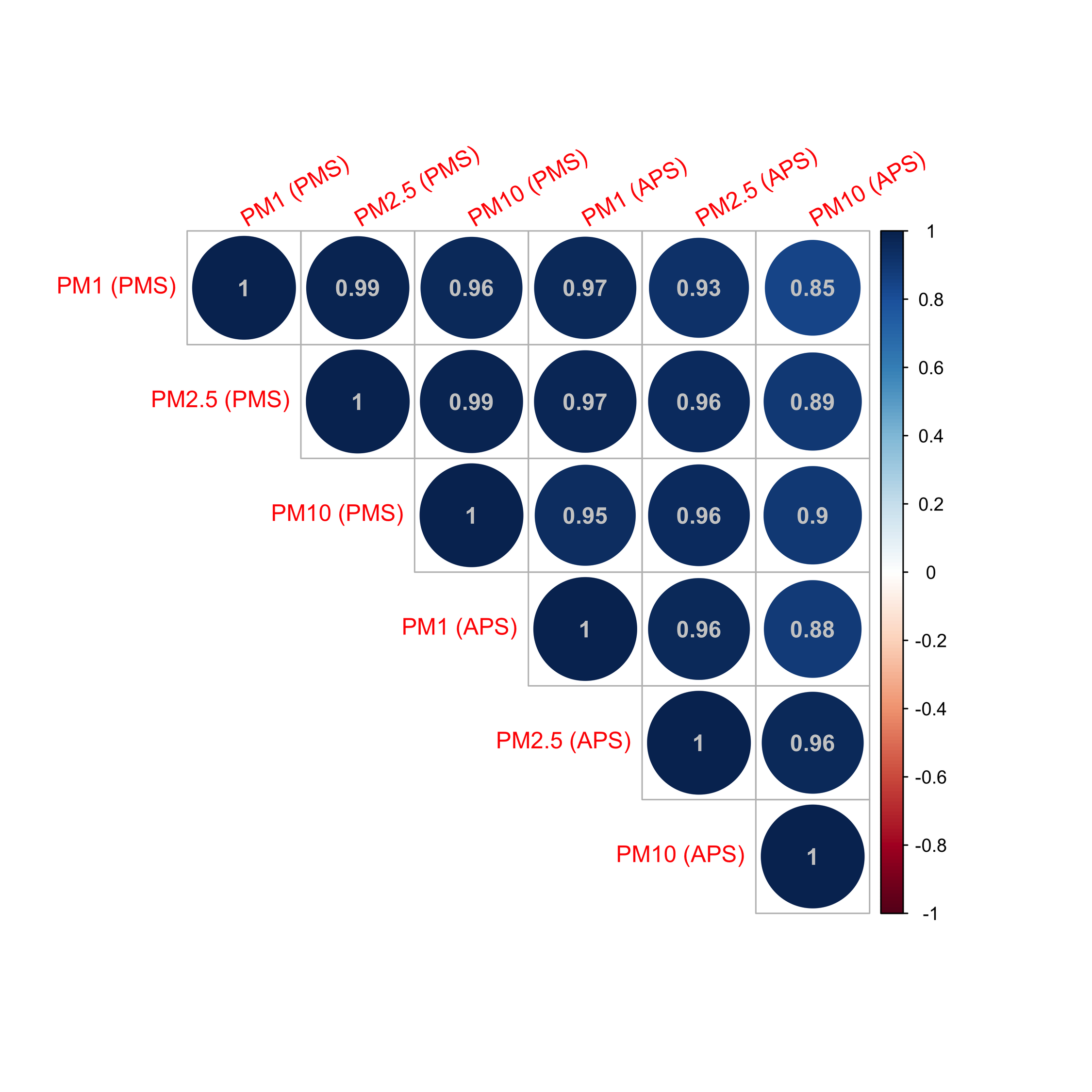

Supplement: S2 Fig — (TIF) [file pone.0259745.s002.tif]

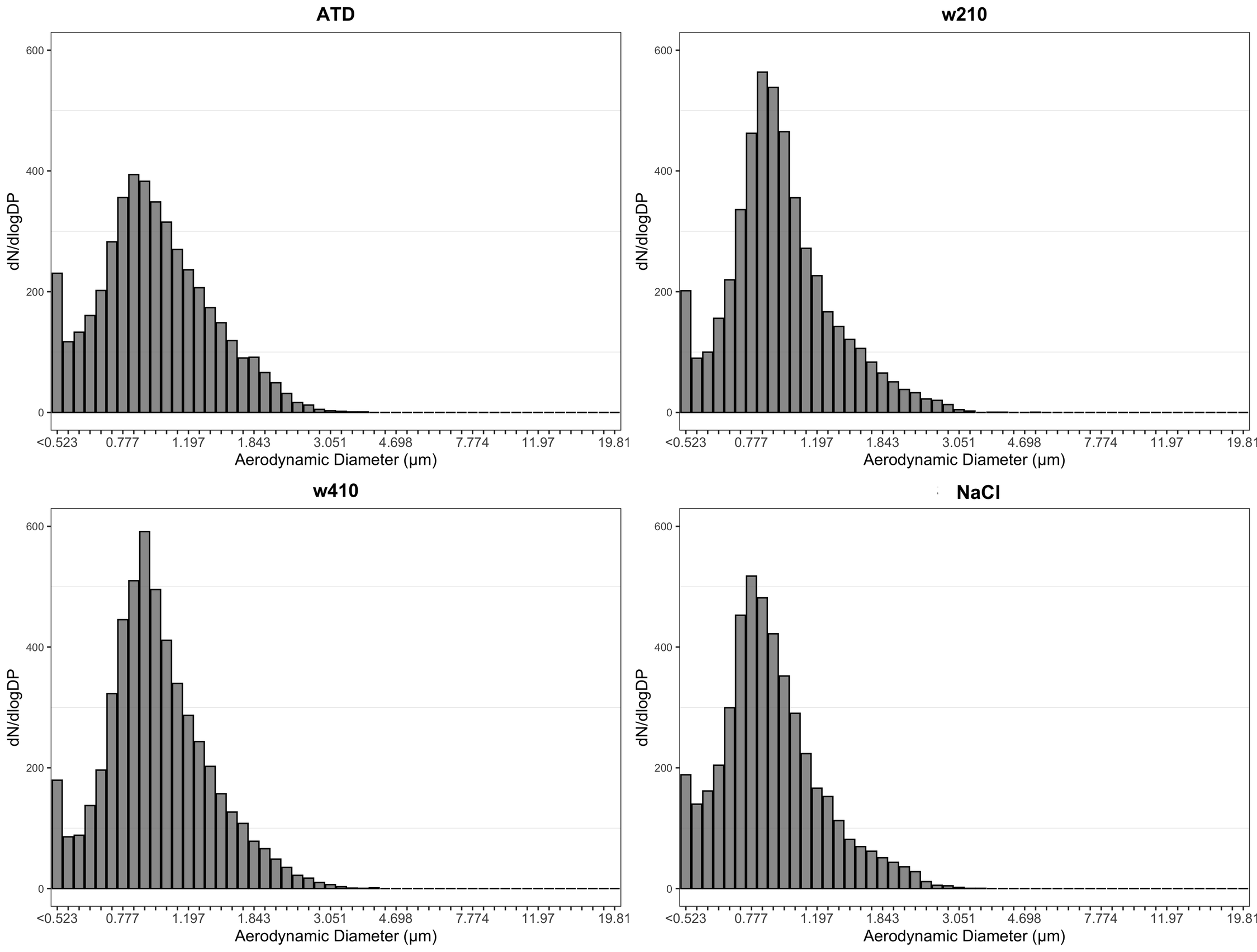

Supplement: S3 Fig — The median diameter of the ATD, saline, W210 and W410 aerosol are 0.94 μm, 0.86 μm, 0.92 μm and 0.96 μm, respectively. (TIF) [file pone.0259745.s003.tif]

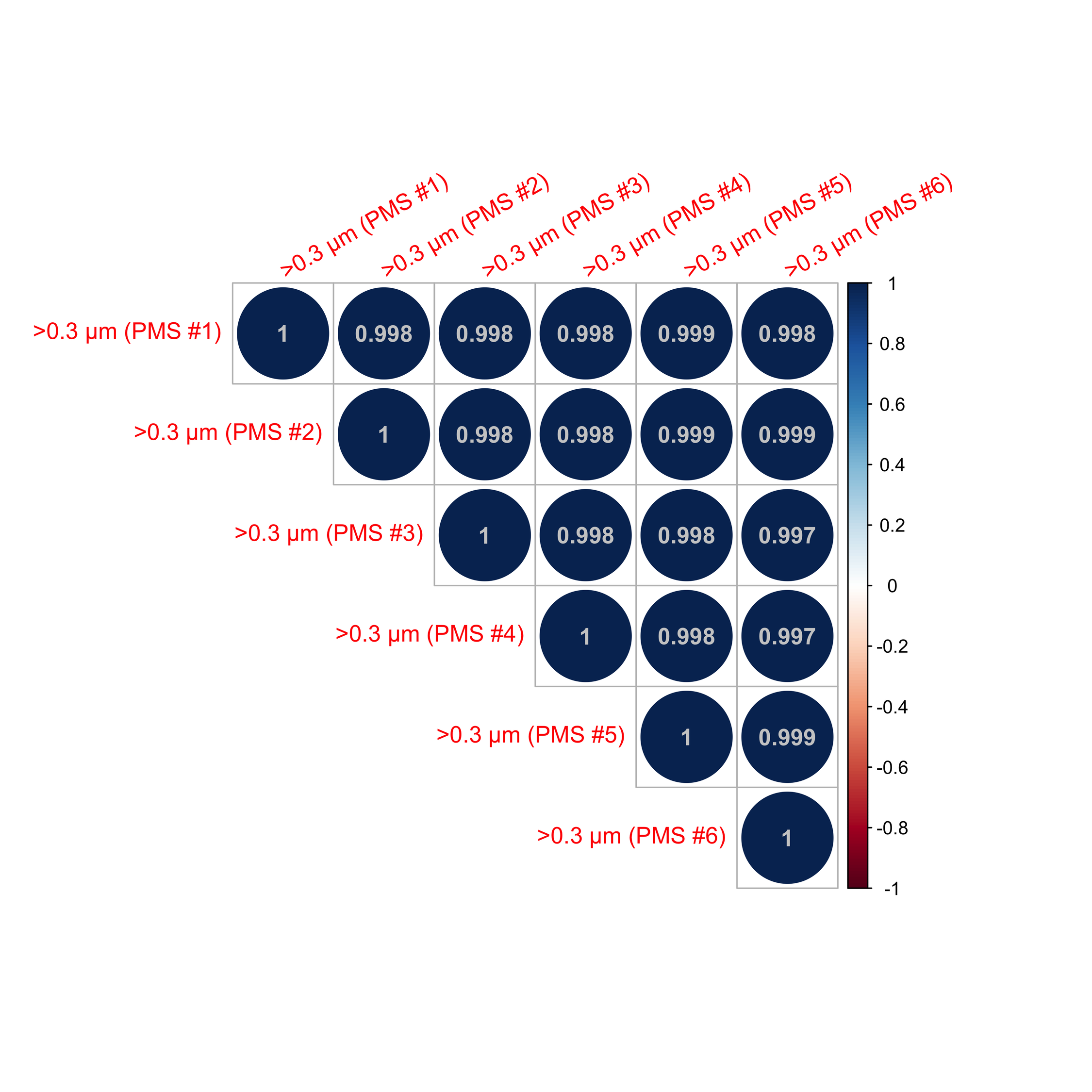

Supplement: S4 Fig — (TIF) [file pone.0259745.s004.tif]

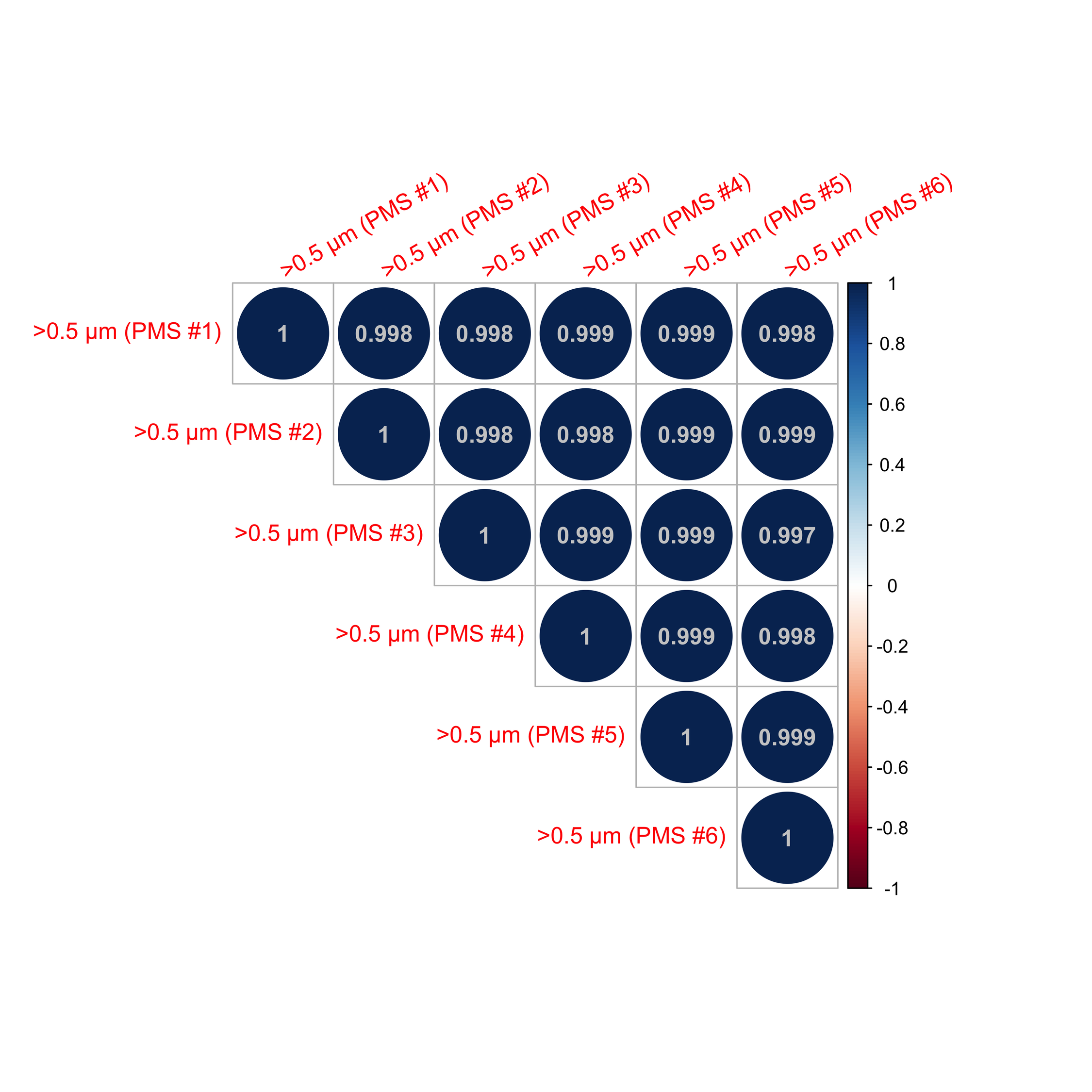

Supplement: S5 Fig — (TIF) [file pone.0259745.s005.tif]

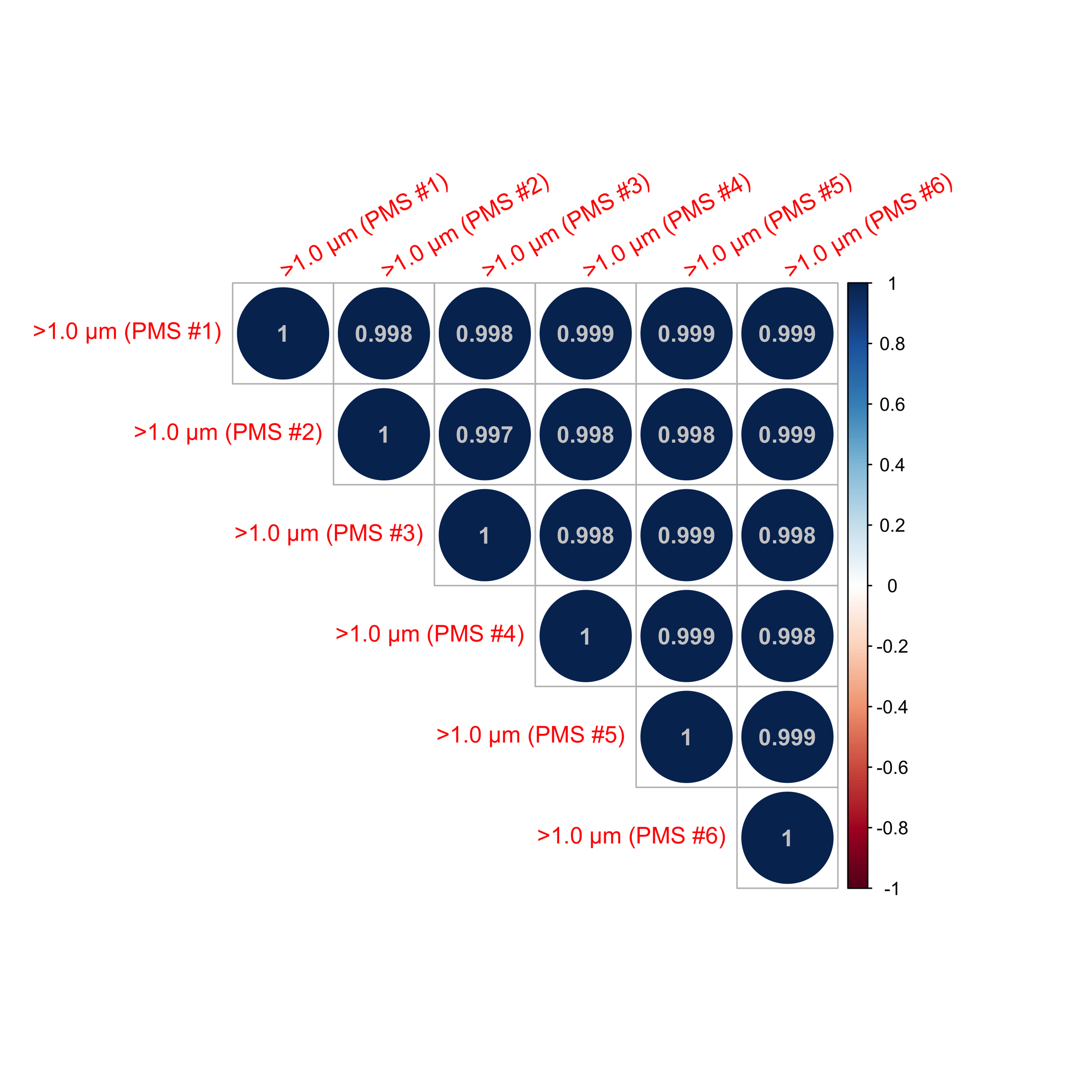

Supplement: S6 Fig — (TIF) [file pone.0259745.s006.tif]

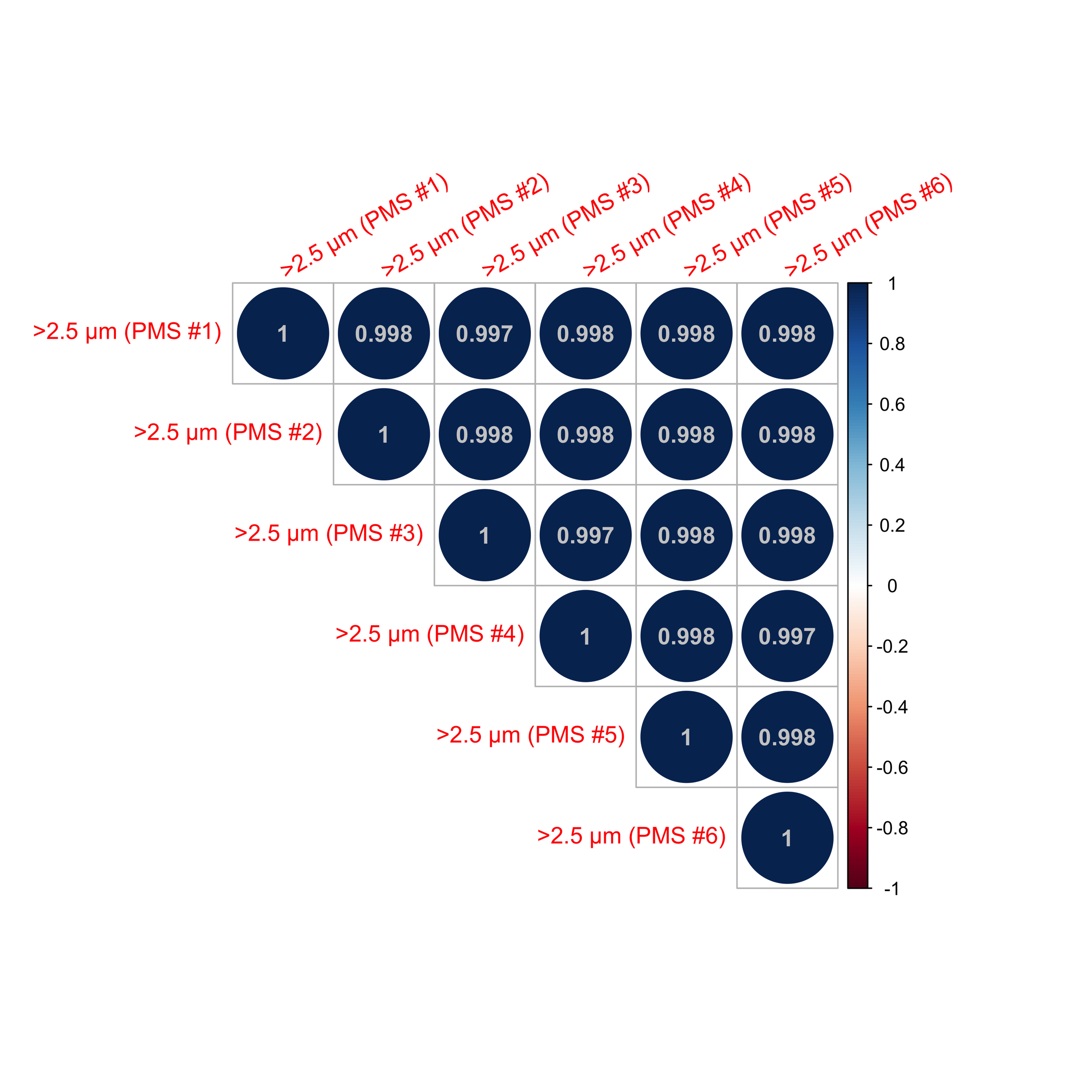

Supplement: S7 Fig — (TIF) [file pone.0259745.s007.tif]

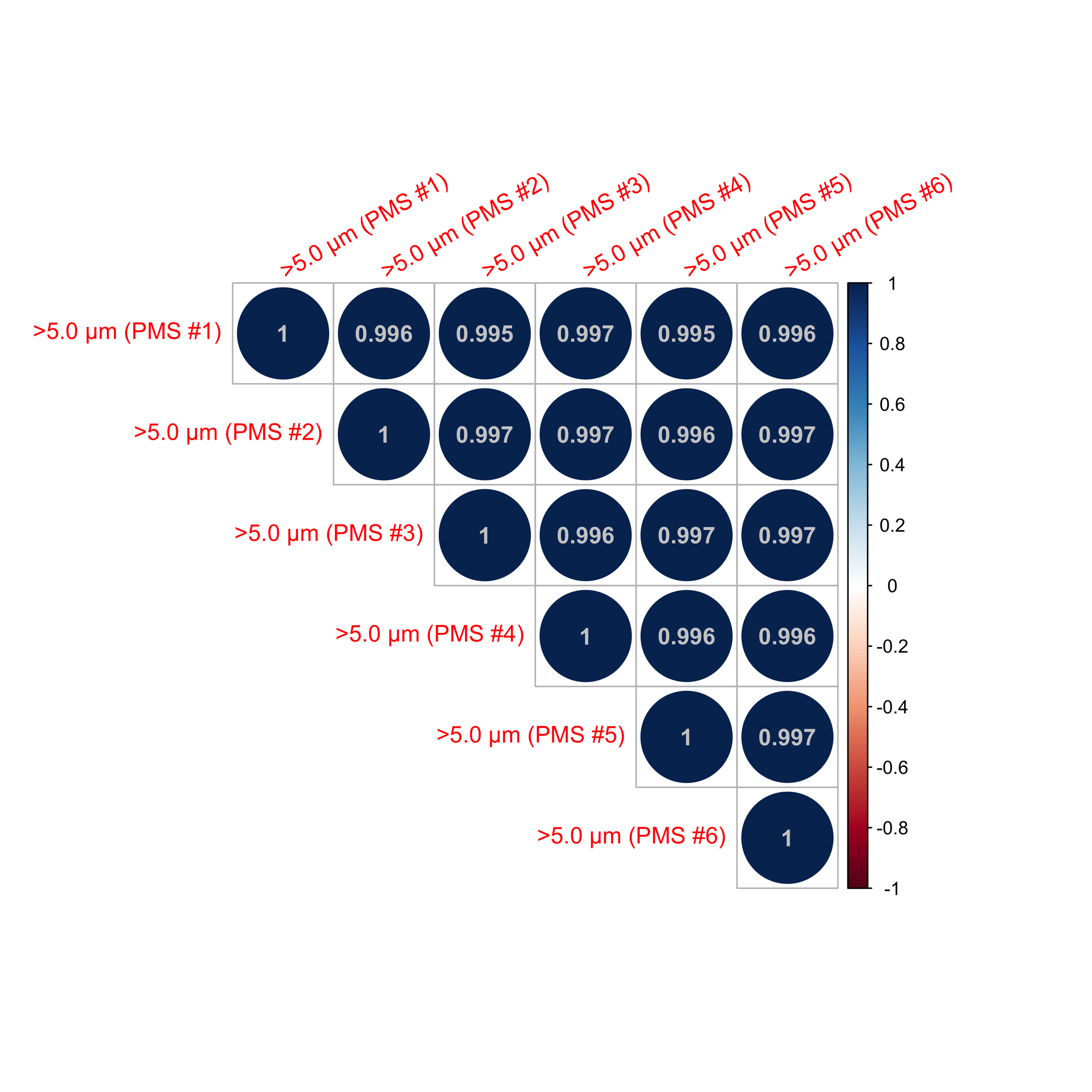

Supplement: S8 Fig — (TIF) [file pone.0259745.s008.tif]

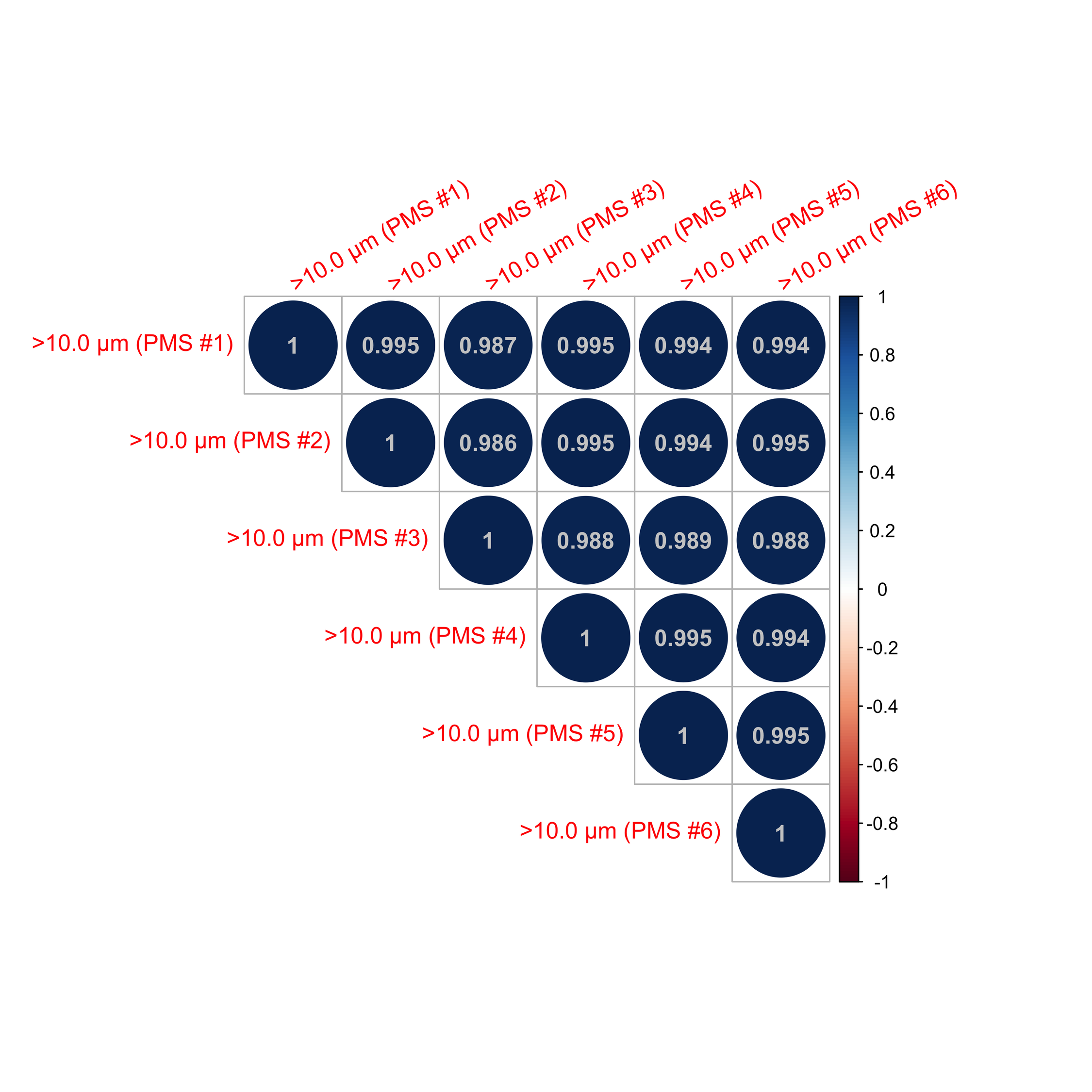

Supplement: S9 Fig — (TIF) [file pone.0259745.s009.tif]

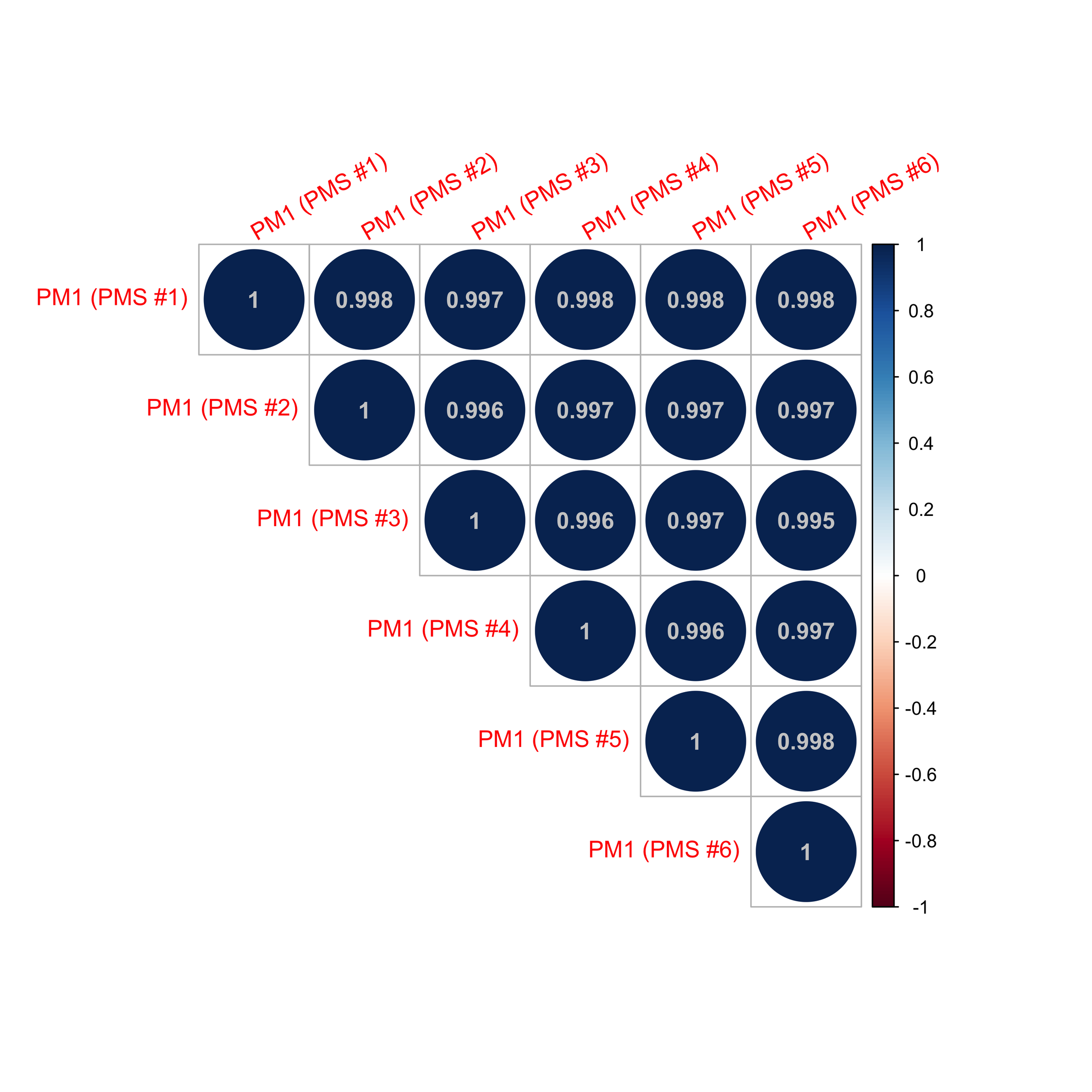

Supplement: S10 Fig — (TIF) [file pone.0259745.s010.tif]

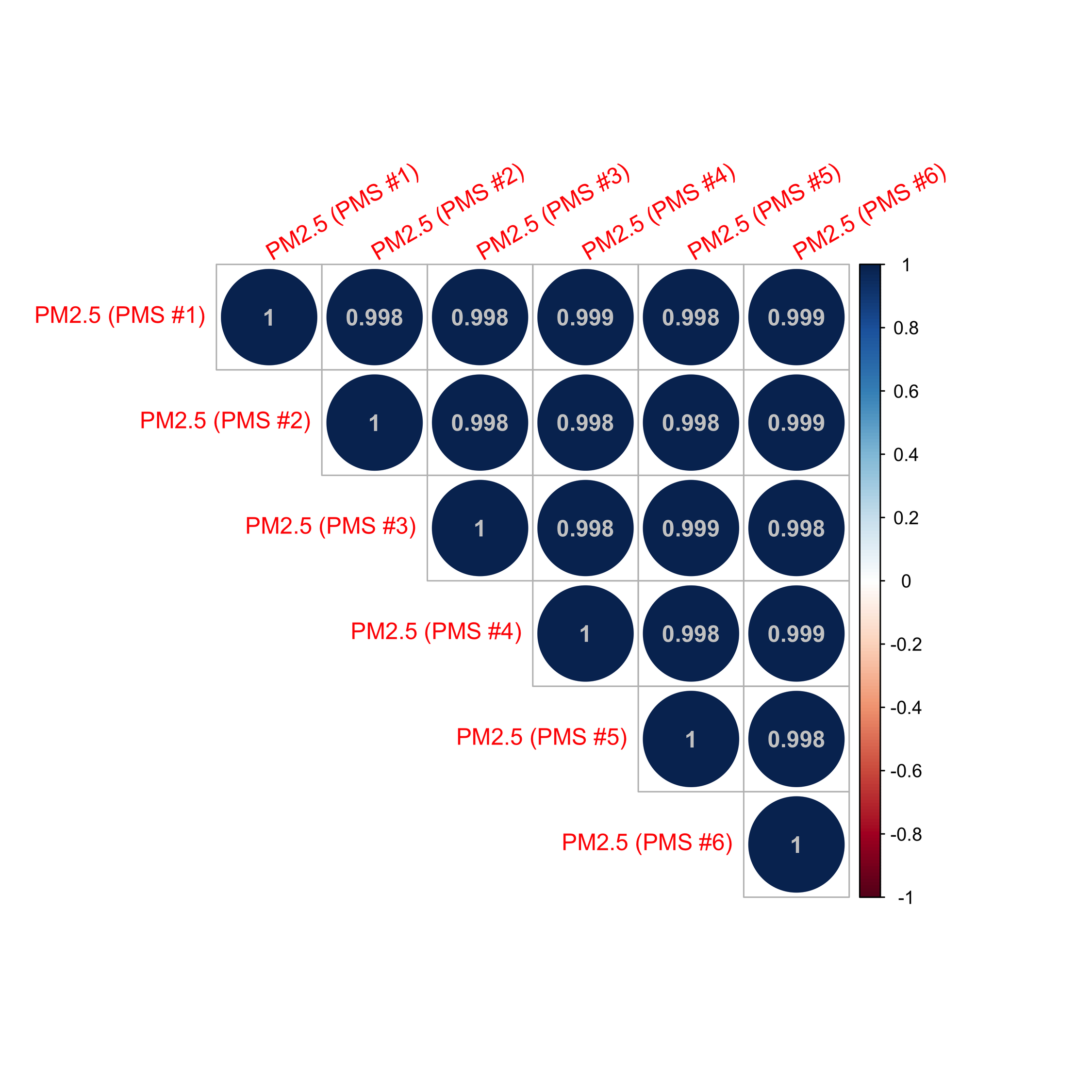

Supplement: S11 Fig — (TIF) [file pone.0259745.s011.tif]

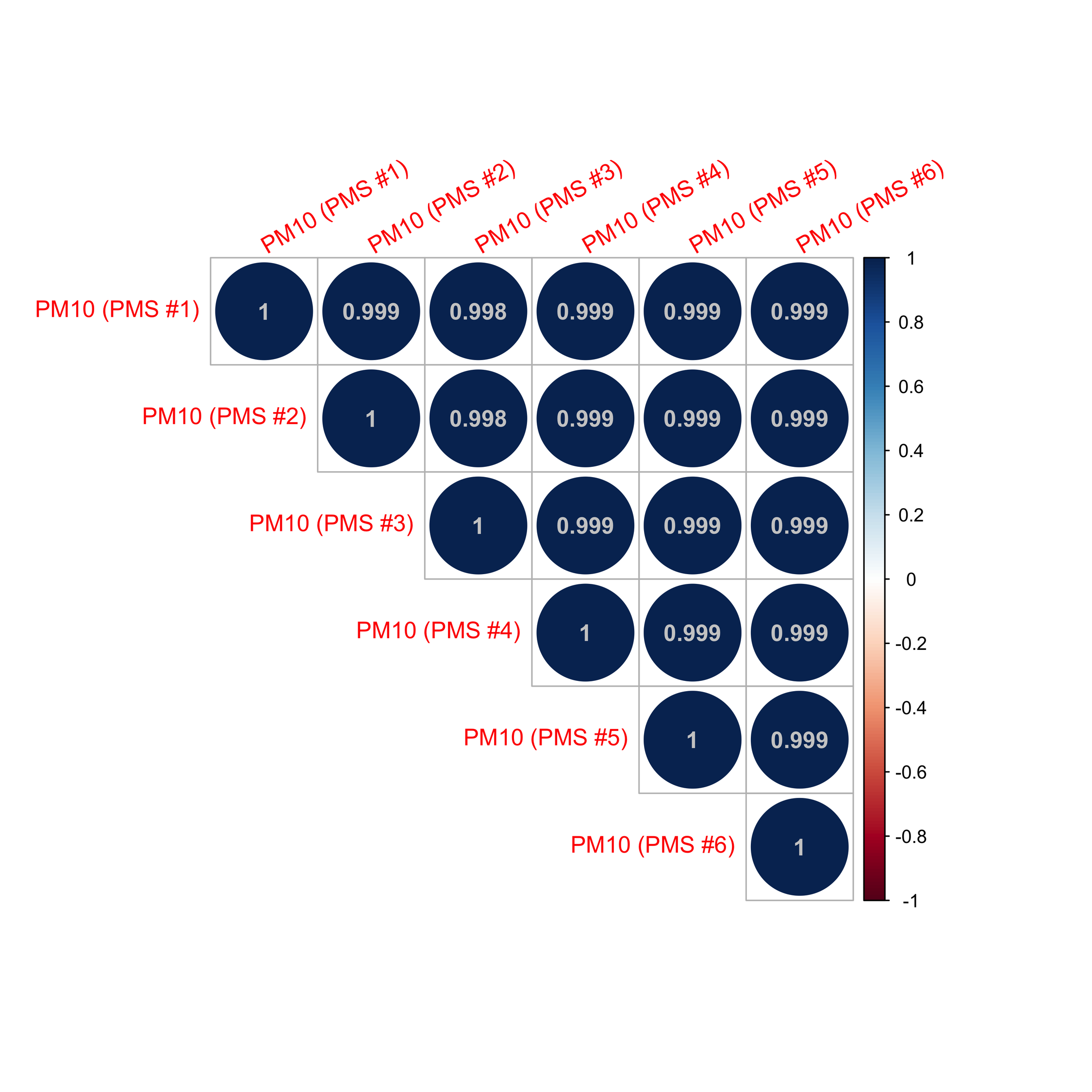

Supplement: S12 Fig — (TIF) [file pone.0259745.s012.tif]

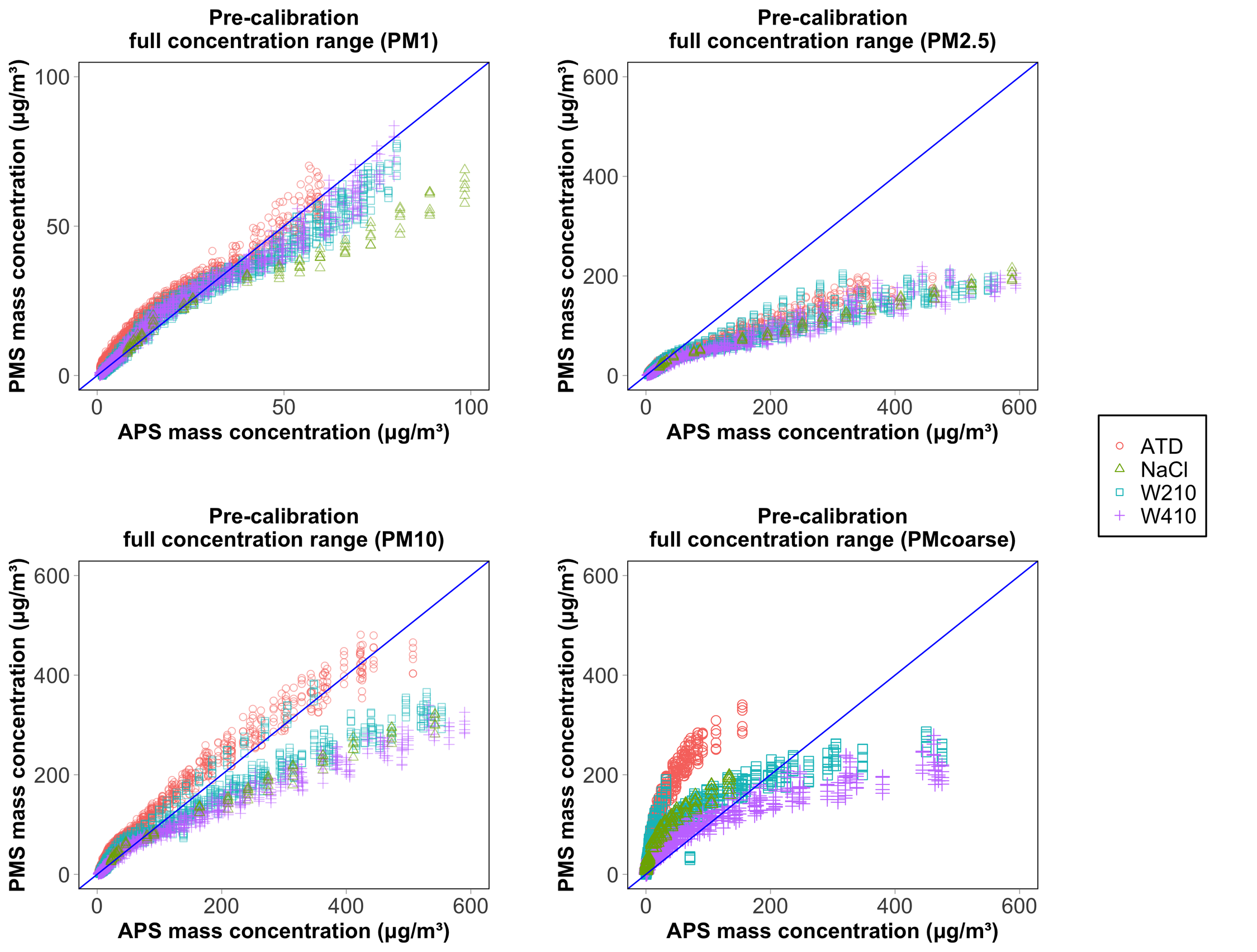

Supplement: S13 Fig — (TIF) [file pone.0259745.s013.tif]

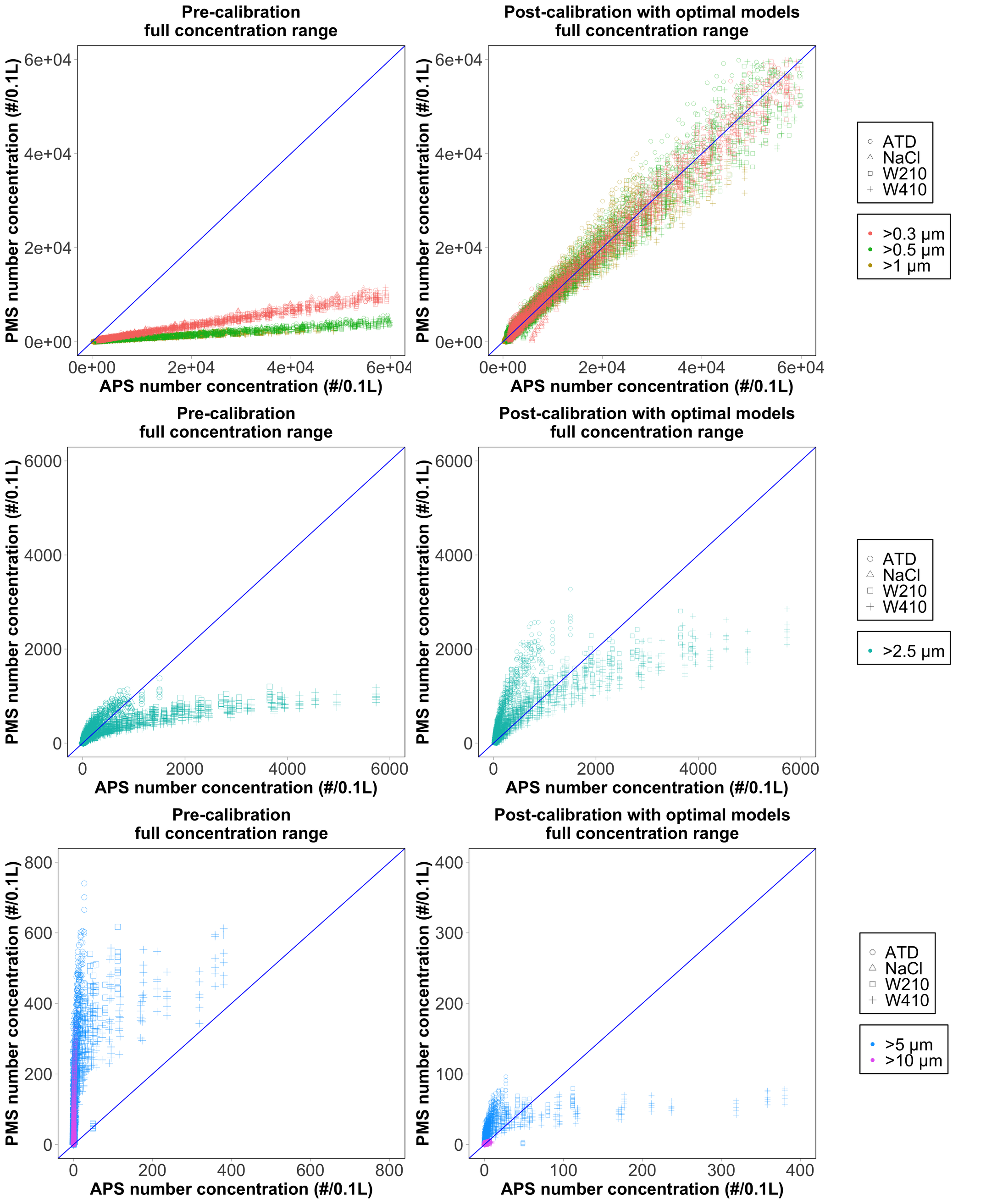

Supplement: S14 Fig — (TIF) [file pone.0259745.s014.tif]
