# Supplementary material for: Assessing the value of complex refractive index and particle density for calibration of low-cost particle matter sensor for size-resolved particle count and PM2.5 measurements
Source: PLoS One. 2021 Nov 11;16(11):e0259745. doi: 10.1371/journal.pone.0259745 (PMC8584671; doi:10.1371/journal.pone.0259745)
Supplement: S1 Table — (DOCX) [file pone.0259745.s015.docx]

**S1 Table. Forms of linear model fitted for number concentration and mass concentration for calibration.**

| **Indices** | **Form of linear model** | **Equation** |
| --- | --- | --- |
| Number  concentration | Linear | $\mathrm{APS}_{t}= \beta_{0}+\beta_{1} \mathrm{PMS}_{t}+\varepsilon_{t}$ |
|  | Linear + CRI | $\mathrm{APS}_{t}= \beta_{0}+\beta_{1} \mathrm{PMS}_{t}+\beta_{2}CRI+\varepsilon_{t}$ |
|  | Linear + RH | $\mathrm{APS}_{t}= \beta_{0}+\beta_{1} \mathrm{PMS}_{t}+\beta_{2}RH+\varepsilon_{t}$ |
|  | Linear + Density | $\mathrm{APS}_{t}= \beta_{0}+\beta_{1} \mathrm{PMS}_{t}+\beta_{2}Density+\varepsilon_{t}$ |
|  | Linear + CRI + Density | $\mathrm{APS}_{t}= \beta_{0}+\beta_{1} \mathrm{PMS}_{t}+\beta_{2}CRI+\beta_{3}Density+\varepsilon_{t}$ |
|  | Linear + CRI + RH | $\mathrm{APS}_{t}= \beta_{0}+\beta_{1} \mathrm{PMS}_{t}+\beta_{2}CRI+\beta_{3}RH+\varepsilon_{t}$ |
| Mass  concentration | Linear | $\mathrm{APS}_{t}= \beta_{0}+\beta_{1} \mathrm{PMS}_{t}+\varepsilon_{t}$ |
|  | Linear + CRI | $\mathrm{APS}_{t}= \beta_{0}+\beta_{1} \mathrm{PMS}_{t}+\beta_{2}CRI+\varepsilon_{t}$ |
|  | Linear + RH | $\mathrm{APS}_{t}= \beta_{0}+\beta_{1} \mathrm{PMS}_{t}+\beta_{2}RH+\varepsilon_{t}$ |
|  | Linear + Density | $\mathrm{APS}_{t}= \beta_{0}+\beta_{1} \mathrm{PMS}_{t}+\beta_{2}Density+\varepsilon_{t}$ |
|  | Linear + CRI + Density | $\mathrm{APS}_{t}= \beta_{0}+\beta_{1} \mathrm{PMS}_{t}+\beta_{2}CRI+\beta_{3}Density+\varepsilon_{t}$ |
|  | Linear + CRI + RH | $\mathrm{APS}_{t}= \beta_{0}+\beta_{1} \mathrm{PMS}_{t}+\beta_{2}CRI+\beta_{3}RH+\varepsilon_{t}$ |
|  | Polynomial | $\mathrm{APS}_{t}= \beta_{0}+\beta_{1} \mathrm{PMS}_{t}+\beta_{2} {\mathrm{PMS}^{2}}_{t}+\varepsilon_{t}$ |
|  | Polynomial + CRI | $\mathrm{APS}_{t}= \beta_{0}+\beta_{1} \mathrm{PMS}_{t}+\beta_{2} {\mathrm{PMS}^{2}}_{t}+\beta_{3}CRI+\varepsilon_{t}$ |
|  | Polynomial + RH | $\mathrm{APS}_{t}= \beta_{0}+\beta_{1} \mathrm{PMS}_{t}+\beta_{2} {\mathrm{PMS}^{2}}_{t}+\beta_{3}RH+\varepsilon_{t}$ |
|  | Polynomial + Density | $\mathrm{APS}_{t}= \beta_{0}+\beta_{1} \mathrm{PMS}_{t}+\beta_{2} {\mathrm{PMS}^{2}}_{t}+\beta_{3}Density+\varepsilon_{t}$ |
|  | Polynomial + CRI + Density | $\mathrm{APS}_{t}= \beta_{0}+\beta_{1} \mathrm{PMS}_{t}+\beta_{2} {\mathrm{PMS}^{2}}_{t}+\beta_{3}CRI+\beta_{4}Density+\varepsilon_{t}$ |
|  | Polynomial + CRI + RH | $\mathrm{APS}_{t}= \beta_{0}+\beta_{1} \mathrm{PMS}_{t}+\beta_{2} {\mathrm{PMS}^{2}}_{t}+\beta_{3}CRI+\beta_{4}RH+\varepsilon_{t}$ |
