# Supplementary material for: Assessing the value of complex refractive index and particle density for calibration of low-cost particle matter sensor for size-resolved particle count and PM2.5 measurements
Source: PLoS One. 2021 Nov 11;16(11):e0259745. doi: 10.1371/journal.pone.0259745 (PMC8584671; doi:10.1371/journal.pone.0259745)
Supplement: S2 Table — (DOCX) [file pone.0259745.s016.docx]

**S2 Table. Summary of the R^2^, Bayesian information criterion (BIC), and the normalized mean absolute error (NMAE) of the**

**calibration models for number concentration.**

| **Indices** | **Equation** | **Regression ^a^** | **R^2^** | **BIC** | **NMAE** |
| --- | --- | --- | --- | --- | --- |
| ***Full concentration range (APS total number concentration between 0 – 1000 #/ cm^3^) (n = 4,134)*** | | | | |  |
| >0.3 µm | Linear | y = 5.93 x | 0.99 | 78723 | 2.20% |
|  | Linear + CRI | y = 6.02 x - 516.26 CRI | 0.99 | 78603 | 2.07% |
|  | Linear + RH | y = 5.99 x - 13.72 RH | 0.99 | 78684 | 2.12% |
|  | Linear + density | y = 6.00 x - 244.83 density | 0.99 | 78659 | 2.09% |
|  | **Linear + CRI + density** | **y = 6.06 x - 7924 CRI + 4709 density** | **0.99** | **78134** | **2.02%** |
|  | Linear + CRI + RH | y = 6.00 x - 1090 CRI + 28.23 RH | 0.99 | 78567 | 2.06% |
| >0.5 µm | Linear | y = 14.17 x | 0.98 | 79716 | 2.92% |
|  | Linear + CRI | y = 14.52 x - 605.95 CRI | 0.98 | 79584 | 2.83% |
|  | Linear + RH | y = 14.39 x - 14.59 RH | 0.98 | 79682 | 2.85% |
|  | Linear + density | y = 14.46 x - 322.64 density | 0.98 | 79618 | 2.83% |
|  | **Linear + CRI + density** | **y = 14.59 x - 5054 CRI + 2828 density** | **0.98** | **79462** | **2.81%** |
|  | Linear + CRI + RH | y = 14.40 x - 1434 CRI + 40.68 RH | 0.98 | 79518 | 2.78% |
| >1 µm | Linear | y = 14.85 x | 0.96 | 76002 | 2.88% |
|  | Linear + CRI | y = 15.15 x - 243.6 CRI | 0.97 | 75950 | 2.91% |
|  | Linear + RH | y = 14.99 x - 4.28 RH | 0.96 | 76000 | 2.89% |
|  | Linear + density | y = 15.08 x - 121.37 density | 0.96 | 75973 | 2.90% |
|  | **Linear + CRI + density** | **y = 15.23 x - 3476 CRI + 2064 density** | **0.97** | **75789** | **2.87%** |
|  | Linear + CRI + RH | y = 14.98 x - 784.93 CRI + 26.40 RH | 0.97 | 75884 | 2.89% |
| >2.5 µm | Linear | y = 2.2 x | 0.66 | 62906 | 3.87% |
|  | Linear + CRI | y = 2.48 x - 86.58 CRI | 0.68 | 62717 | 3.95% |
|  | Linear + RH | y = 2.43 x - 2.79 RH | 0.67 | 62808 | 3.84% |
|  | Linear + density | y = 2.46 x - 52.33 density | 0.67 | 62737 | 3.93% |
|  | Linear + CRI + density | y = 2.48 x - 343.19 CRI + 164.55 density | 0.68 | 62699 | 3.93% |
|  | **Linear + CRI + RH** | **y = 2.42 x - 156.71 CRI + 3.38 RH** | **0.68** | **62695** | **3.95%** |
| >5 µm | Linear | y = 0.11 x | 0.31 | 38958 | 2.71% |
|  | Linear + CRI | y = 0.14 x - 3.72 CRI | 0.33 | 38843 | 2.83% |
|  | Linear + RH | y = 0.14 x - 0.16 RH | 0.32 | 38849 | 2.78% |
|  | Linear + density | y = 0.14 x - 2.27 density | 0.32 | 38853 | 2.82% |
|  | **Linear + CRI + density** | **y = 0.13 x - 12.76 CRI + 5.81 density** | **0.33** | **38841** | **2.82%** |
|  | Linear + CRI + RH | y = 0.14 x - 2.41 CRI - 0.06 RH | 0.33 | 38848 | 2.83% |
| >10 µm | Linear | y = 0.11 x | 0.70 | 8117 | 3.66% |
|  | Linear + CRI | y = 0.14 x - 3.72 CRI | 0.71 | 7997 | 3.69% |
|  | Linear + RH | y = 0.14 x - 0.16 RH | 0.71 | 7998 | 3.65% |
|  | Linear + density | y = 0.14 x - 2.27 density | 0.71 | 8022 | 3.66% |
|  | **Linear + CRI + density** | **y = 0.13 x - 12.76 CRI + 5.81 density** | **0.72** | **7918** | **3.67%** |
|  | Linear + CRI + RH | y = 0.14 x - 2.41 CRI - 0.06 RH | 0.71 | 8000 | 3.68% |
| ***Lower concentration range (APS total number concentration < 100 #/ cm^3^) (n = 1,838)*** | | | | | |
| >0.3 µm | Linear | y = 4.84 x | 0.97 | 30263 | 7.96% |
|  | Linear + CRI | y = 4.95 x - 86.88 CRI | 0.97 | 30263 | 8.02% |
|  | Linear + RH | y = 4.80 x - 1.08 RH | 0.97 | 30270 | 7.94% |
|  | Linear + density | y = 4.92 x - 43.29 Density | 0.97 | 30265 | 8.01% |
|  | Linear + CRI + density | y = 4.98 x - 723.33 CRI + 396.15 Density | 0.97 | 30259 | 7.98% |
|  | **Linear + CRI + RH** | **y = 4.94 x - 358.12 CRI + 13.68 RH** | **0.97** | **30235** | **7.94%** |
| >0.5 µm | Linear | y = 10.84 x | 0.93 | 30285 | 10.39% |
|  | Linear + CRI | y = 10.89 x - 9.97 CRI | 0.93 | 30293 | 10.41% |
|  | Linear + RH | y = 10.38 x + 5.12 RH | 0.93 | 30276 | 10.21% |
|  | Linear + density | y = 10.89 x - 5.98 Density | 0.93 | 30293 | 10.41% |
|  | Linear + CRI + density | y = 10.89 x - 15.80 CRI + 3.63 density | 0.93 | 30300 | 10.41% |
|  | **Linear + CRI + RH** | **y = 10.83 x - 385.61 CRI + 18.91 RH** | **0.94** | **30234** | **10.23%** |
| >1 µm | Linear | y = 12.50 x | 0.92 | 26963 | 8.50% |
|  | Linear + CRI | y = 11.21 x + 105.13 CRI | 0.93 | 26844 | 7.99% |
|  | Linear + RH | y = 10.88 x + 6.37 RH | 0.93 | 26734 | 7.68% |
|  | Linear + density | y = 11.17 x + 69.72 density | 0.93 | 26828 | 7.97% |
|  | Linear + CRI + density | y = 11.33 x - 361.34 CRI + 294.27 density | 0.93 | 26812 | 7.94% |
|  | **Linear + CRI + RH** | **y = 11.10 x - 105.04 CRI + 10.59 RH** | **0.93** | **26713** | **7.57%** |
| >2.5 µm | Linear | y = 0.51 x | 0.60 | 14298 | 7.73% |
|  | Linear + CRI | y = 0.46 x + 1.20 CRI | 0.60 | 14284 | 7.72% |
|  | Linear + RH | y = 0.41 x + 0.13 RH | 0.62 | 14192 | 7.76% |
|  | Linear + density | y = 0.46 x + 0.80 density | 0.60 | 14282 | 7.72% |
|  | Linear + CRI + density | y = 0.46 x - 4.61 CRI + 3.71 density | 0.60 | 14286 | 7.68% |
|  | **Linear + CRI + RH** | **y = 0.45 x - 8.38 CRI + 0.48 RH** | **0.66** | **14018** | **7.22%** |
| >5 µm | Linear | y = 0.01 x | 0.35 | -2612 | 11.52% |
|  | Linear + CRI | y = 0.003 x + 0.04 CRI | 0.43 | -2845 | 12.04% |
|  | **Linear + RH** | **y = 0.004 x + 0.002 RH** | **0.44** | **-2876** | **11.89%** |
|  | Linear + density | y = 0.003 x + 0.03 density | 0.43 | -2856 | 12.01% |
|  | Linear + CRI + density | y - 0.003 x - 0.06 CRI + 0.06 density | 0.44 | -2855 | 11.95% |
|  | Linear + CRI + RH | y = 0.003 x + 0.007 CRI + 0.002 RH | 0.44 | -2869 | 11.91% |
| >10 µm | Linear | y = 0.004 x | 0.19 | -5846 | 15.16% |
|  | Linear + CRI | y = 0.002 x + 0.009 CRI | 0.22 | -5922 | 17.97% |
|  | **Linear + RH** | **y = 0.002 x + 0.0004 RH** | **0.22** | **-5926** | **17.74%** |
|  | Linear + density | y = 0.002 x + 0.006 density | 0.22 | -5922 | 17.95% |
|  | Linear + CRI + density | y = 0.002 x + 0.004 CRI + 0.003 density | 0.22 | -5915 | 17.97% |
|  | Linear + CRI + RH | y = 0.002 x + 0.003 CRI + 0.0003 RH | 0.22 | -5920 | 17.91% |

^a^ y: APS measurement; x: PMS measurement. The models emboldened for each size bin were the optimal model selected according to the BIC.

Definition of abbreviations: n = number of datapoints; CRI = complex index of refraction; RH = relative humidity; BIC = Bayesian information criteria; NMAE = normalized mean absolute error.
