# Supplementary material for: Assessing the value of complex refractive index and particle density for calibration of low-cost particle matter sensor for size-resolved particle count and PM2.5 measurements
Source: PLoS One. 2021 Nov 11;16(11):e0259745. doi: 10.1371/journal.pone.0259745 (PMC8584671; doi:10.1371/journal.pone.0259745)
Supplement: S3 Table — (DOCX) [file pone.0259745.s017.docx]

**S3 Table. Summary of the R^2^, Bayesian information criterion (BIC), and the normalized mean absolute error (NMAE) of the**

**calibration models for mass concentration.**

| **Indices** | **Equation** | **Regression ^a^** | **R^2^** | **BIC ^b^** | **NMAE** |
| --- | --- | --- | --- | --- | --- |
| ***Full concentration range (APS total number concentration between 0 – 1000 #/ cm^3^) (n = 4,134)*** | | | | | |
| PM_1_ | Linear | y = 1.06 x | 0.96 | 25852 | 3.11% |
|  | Polynomial | y = 0.76 x + 0.007 x^2^ | 0.97 | 24480 | 2.41% |
|  | Linear + CRI | y = 1.16 x - 2.12 CRI | 0.97 | 25137 | 2.99% |
|  | Polynomial + CRI | y = 0.82 x + 0.006 x^2^ - 0.58 CRI | 0.97 | 24446 | 2.48% |
|  | Linear + RH | y = 1.15 x - 0.08 RH | 0.97 | 25333 | 3.03% |
|  | Polynomial + RH | y = 0.79 x + 0.01 x^2^ - 0.02 RH | 0.97 | 24471 | 2.46% |
|  | Linear + density | y = 1.16 x - 1.51 density | 0.97 | 24906 | 3.00% |
|  | Polynomial + density | y = 0.87 x + 0.01 x x^2^ - 0.65 density | 0.97 | 24351 | 2.54% |
|  | Linear + CRI + density | y = 1.13 x + 13.88 CRI - 10.13 density | 0.97 | 24181 | 2.84% |
|  | **Polynomial + CRI + density** | **y = 0.83 x + 0.01 x^2^ + 14.44 CRI - 9.58 density** | **0.98** | **23432** | **2.33%** |
|  | Linear + CRI + RH | y = 1.15 x - 2.14 CRI + 0.001 RH | 0.97 | 25143 | 2.99% |
|  | Polynomial + CRI + RH | y = 0.82 x + 0.006 x^2^ - 0.78 CRI + 0.01 RH | 0.97 | 24451 | 2.47% |
| PM_2.5_ | Linear | y = 2.29 x | 0.94 | 42435 | 4.53% |
|  | Polynomial | y = 1.55 x + 0.006 x^2^ | 0.96 | 41341 | 3.41% |
|  | Linear + CRI | y = 2.54 x - 16.04 CRI | 0.95 | 41613 | 4.07% |
|  | Polynomial + CRI | y = 1.86 x + 0.004 x^2^ - 7.36 CRI | 0.96 | 41227 | 3.47% |
|  | Linear + RH | y = 2.50 x - 0.53 RH | 0.95 | 41983 | 4.12% |
|  | Polynomial + RH | y = 1.64 x + 0.005 x^2^ - 0.1 RH | 0.96 | 41337 | 3.41% |
|  | Linear + density | y = 2.53 x - 10.36 density | 0.95 | 41568 | 4.05% |
|  | Polynomial + density | y = 1.88 x + 0.004 x^2^ - 5.11 density | 0.96 | 41199 | 3.48% |
|  | Linear + CRI + density | y = 2.52 x + 13.55 CRI - 18.82 density | 0.95 | 41565 | 4.04% |
|  | Polynomial + CRI + density | y = 1.85 x + 0.004 x^2^ + 22.74 CRI - 19.13 density | 0.96 | 41172 | 3.44% |
|  | Linear + CRI + RH | y = 2.51 x - 23.27 CRI + 0.36 RH | 0.95 | 41565 | 4.07% |
|  | **Polynomial + CRI + RH** | **y = 1.80 x + 0.004 x^2^ - 15.55 CRI + 0.42 RH** | **0.96** | **41152** | **3.44%** |
| PM_10_ | Linear | y = 1.53 x | 0.85 | 49963 | 3.56% |
|  | Polynomial | y = 0.72 x - 0.003 x^2^ | 0.88 | 48959 | 2.61% |
|  | Linear + CRI | y = 1.72 x - 27.80 CRI | 0.86 | 49555 | 3.31% |
|  | Polynomial + CRI | y = 0.78 x + 0.003 x^2^ - 3.13 CRI | 0.88 | 48963 | 2.63% |
|  | Linear + RH | y = 1.69 x - 0.96 RH | 0.86 | 49716 | 3.33% |
|  | Polynomial + RH | y = 0.66 x - 0.003 x^2^ + 0.13 RH | 0.88 | 48963 | 2.62% |
|  | Linear + density | y = 1.71 x - 17.91 density | 0.86 | 49541 | 3.32% |
|  | Polynomial + density | y = 0.80 x - 0.003 x^2^ - 2.9 density | 0.88 | 48958 | 2.65% |
|  | Linear + CRI + density | y = 1.71 x + 12.33 CRI - 25.67 density | 0.86 | 49548 | 3.32% |
|  | Polynomial + CRI + density | y = 0.75 x + 0.003 x^2^ + 55.58 CRI - 37.27 density | 0.88 | 48934 | 2.62% |
|  | Linear + CRI + RH | y = 1.69 x - 39.14 CRI + 0.56 RH | 0.87 | 49544 | 3.31% |
|  | **Polynomial + CRI + RH** | **y = 0.73 x + 0.003 x^2^ - 17.94 CRI + 0.75 RH** | **0.88** | **48931** | **2.61%** |
| ***Lower concentration (APS total number concentration < 100 #/ cm^3^) (n = 1,838)*** | | | | | |
| PM_1_ | Linear | y = 0.72 x | 0.90 | 6211 | 10.10% |
|  | Polynomial | y = 0.91 x - 0.02 x^2^ | 0.91 | 6053 | 9.23% |
|  | Linear + CRI | y = 0.6 x + 0.50 CRI | 0.91 | 6020 | 8.82% |
|  | Polynomial + CRI | y = 0.71 x - 0.01 x^2^ + 0.36 CRI | 0.91 | 6017 | 8.82% |
|  | Linear + RH | y = 0.60 x + 0.02 RH | 0.92 | 5979 | 8.75% |
|  | Polynomial + RH | y = 0.70 x - 0.001 x^2^ + 0.02 RH | 0.92 | 5971 | 8.68% |
|  | Linear + density | y = 0.62 x + 0.26 density | 0.91 | 6077 | 9.04% |
|  | Polynomial + density | y = 0.82 x - 0.02 x^2^ + 0.1 density | 0.91 | 6052 | 9.06% |
|  | Linear + CRI + density | y = 0.57 x + 4.80 CRI - 2.68 density | 0.93 | 5746 | 8.16% |
|  | **Polynomial + CRI + density** | **y = 0.80 x - 0.02 x^2^ + 4.93 CRI - 2.95 density** | **0.93** | **5694** | **8.08%** |
|  | Linear + CRI + RH | y = 0.60 x + 0.08 CRI + 0.02 RH | 0.92 | 5989 | 8.73% |
|  | Polynomial + CRI + RH | y = 0.72 x - 0.01 x^2^ - 0.1 CRI + 0.02 RH | 0.92 | 5987 | 8.69% |
| PM_2.5_ | Linear | y = 1.10 x | 0.91 | 11170 | 9.14% |
|  | Polynomial | y = 1.34 x - 0.01 x^2^ | 0.91 | 11087 | 8.80% |
|  | Linear + CRI | y = 0.98 x + 1.37 CRI | 0.92 | 11061 | 8.48% |
|  | Polynomial + CRI | y = 1.09 x - 0.004 x^2^ + 1.06 CRI | 0.92 | 11064 | 8.49% |
|  | Linear + RH | y = 0.94 x + 0.09 RH | 0.92 | 10932 | 8.11% |
|  | Polynomial + RH | y = 0.92 x + 0.001 x^2^ + 0.09 RH | 0.92 | 10939 | 8.11% |
|  | Linear + density | y = 0.99 x + 0.80 density | 0.91 | 11076 | 8.55% |
|  | Polynomial + density | y = 1.14 x - 0.01 x^2^ + 0.54 density | 0.92 | 11074 | 8.58% |
|  | Linear + CRI + density | y = 0.97 x + 6.51 CRI - 3.23 density | 0.92 | 11043 | 8.40% |
|  | Polynomial + CRI + density | y = 1.09 x - 0.004 x^2^ + 6.28 CRI - 3.31 density | 0.92 | 11045 | 8.42% |
|  | Linear + CRI + RH | y = 0.97 x -1.87 CRI + 0.16 RH | 0.92 | 10890 | 8.01% |
|  | **Polynomial + CRI + RH** | **y = 1.14 x - 0.006 x^2^ - 2.43 CRI + 0.17 RH** | **0.92** | **10885** | **7.97%** |
| PM_10_ | Linear | y = 0.63 x | 0.89 | 11878 | 9.30% |
|  | Polynomial | y = 0.86 x - 0.01 x^2^ | 0.90 | 11686 | 8.53% |
|  | Linear + CRI | y = 0.55 x + 1.92 CRI | 0.90 | 11716 | 8.45% |
|  | Polynomial + CRI | y = 0.75 x - 0.004 x^2^ + 0.88 CRI | 0.90 | 11675 | 8.34% |
|  | Linear + RH | y = 0.52 x + 0.12 RH | 0.91 | 11566 | 8.10% |
|  | Polynomial + RH | y = 0.62 x - 0.002 x^2^ + 0.1 RH | 0.91 | 11556 | 7.99% |
|  | Linear + density | y = 0.55 x + 1.12 density | 0.90 | 11741 | 8.54% |
|  | Polynomial + density | y = 0.78 x - 0.004 x^2^ + 0.38 density | 0.90 | 11685 | 8.41% |
|  | Linear + CRI + density | y = 0.54 x - 11.26 CRI - 5.92 density | 0.90 | 11662 | 8.26% |
|  | Polynomial + CRI + density | y = 0.72 x - 0.003 x^2^ + 9.58 CRI - 5.45 density | 0.90 | 11631 | 8.16% |
|  | Linear + CRI + RH | y = 0.54 x - 2.15 CRI + 0.20 RH | 0.91 | 11627 | 8.01% |
|  | **Polynomial + CRI + RH** | **y = 0.78 x - 0.004 x^2^ - 3.57 CRI + 0.21 RH** | **0.91** | **11461** | **7.75%** |

^a^ y: APS measurement; x: PMS measurement. The models emboldened for each size bin were the optimal model selected according to the BIC.

Definition of abbreviations: n = number of datapoints; CRI = complex index of refraction; RH = relative humidity; BIC = Bayesian information criteria; NMAE = normalized mean absolute error.
