# Supplementary material for: Assessing the value of complex refractive index and particle density for calibration of low-cost particle matter sensor for size-resolved particle count and PM2.5 measurements
Source: PLoS One. 2021 Nov 11;16(11):e0259745. doi: 10.1371/journal.pone.0259745 (PMC8584671; doi:10.1371/journal.pone.0259745)
Supplement: Attachment — Submitted filename: Response to reviewers.pdf [file pone.0259745.s019.pdf]

***PloS One* Peer Review Comments and Responses**

September 18, 2021

We would like to thank the Editor and Reviewers for their thoughtful comments on how we can improve our manuscript. We reproduce each comment and provide responses below. Additional grammar check was done to improve readability of the manuscript.

Comments from the Editor
Thank you for submitting your manuscript to PLOS ONE. After careful consideration, we feel that it has merit but does not fully meet PLOS ONE's publication criteria as it currently stands. Therefore, we invite you to submit a revised version of the manuscript that addresses the points raised during the review process.

Thank you for revising the manuscript. This is much improved.

Please note that I did not send this out for re-review. Instead I reviewed the whole manuscript and the responses carefully myself. Please find my detailed comments (at the end of this letter) for you to address before I can recommend for publication in PlosOne.

Please submit your revised manuscript by 18 Sep. If you will need more time than this to complete your revisions, please reply to this message or contact the journal office at plosone@plos.org. When you're ready to submit your revision, log on to https://www.editorialmanager.com/pone/ and select the 'Submissions Needing Revision' folder to locate your manuscript file.

Please include the following items when submitting your revised manuscript:

A rebuttal letter that responds to each point raised by the academic editor and reviewer(s). You should upload this letter as a separate file labeled 'Response to Reviewers'.

A marked-up copy of your manuscript that highlights changes made to the original version. You should upload this as a separate file labeled 'Revised Manuscript with Track Changes'.

An unmarked version of your revised paper without tracked changes. You should upload this as a separate file labeled 'Manuscript'.

If you would like to make changes to your financial disclosure, please include your updated statement in your cover letter. Guidelines for resubmitting your figure files are available below the reviewer comments at the end of this letter.

Additional Editor Comments
1. Response to Q1 by reviewer 2: I believe the reviewer is asking for the lower size cut of APS and PMS. If you look at the specification of the APS you should be able to find out the lower size limit with 50% efficiency. The lower size limit for optical detection is 370nm. In the

manuscript you mentioned that size ranges from 0.5 um to 20um. It should be noted that this is not an optical size so not comparable to PPS which is optical diameter.

Reply: Thank you for the comments. We added the discussion about the optical size and aerodynamic size of the APS. We clarified the focus of our study in the last paragraph of the "Plantower PMS A003 and Sensor Test Platform" section.

pp 9, lines 152 – 165 in the revised text:
"The reference instrument used in this study is the TSI Aerodynamic Particle Sizer (APS) 3321 spectrometer. APS measures both the aerodynamic size and optical size of a particle. Using the time-of-flight principle, the APS measures size-resolved particle counts with aerodynamic particle diameter (AD) of 0.523 to 20 µm in 52 size bins. The lower detection limit for optical size is 0.37 µm. Particles with AD<0.523 µm are detected by the APS's optical sensor, but they can not be resolved based on their aerodynamic size. Thus these particles are assigned to a single bin <0.523 µm. The aerodynamic size determined the particle's aerodynamic behavior, such as settling velocity or penetration into the respiratory tract. Thus, we evaluate the correlation between the PMS number concentrations and the APS aerodynamic size bin number concentrations. The instrument estimates mass concentration by assuming spherical particles and particle density input. APS reports particle concentrations with a 5-second resolution. In the experiments, the sampling inlet was placed near the PMS sensors. Per APS specifications, the maximum recommended particle concentration is 1000 $\#/cm^3$ at 0.5 µm with < 5% coincidence. Therefore, the total number concentration of the aerosols in the test chamber was maintained below 1000 #/cc (10^5 #/0.1L)."

2. Figure 4 clearly demonstrate the major difference due to the size cut. Is it more meaningful to use the actual number concentration rather than percentage for this figure. The reason is that if you look at ATD, you can see that the fraction of 0.5-1um is nearly 50% for APS but less than 20% for PMS – this is because the number concentration of particles from 0.3-0.5 um is much higher than 0.37-0.52um size range from APS. Please consider this and respond to the comment.

Reply: Thank you for your suggestion. We agree that it's more meaningful to show the number concentration than the percentage. We revised Figure 4 and the corresponding text.

Revised Figure 4:

Fig 4. A comparison of the size distribution measured by the APS and PMS using OEM calibration. Data from one of the six PMS for ATD, NaCl, W210, and W410 particles taken 15 minutes after the aerosols were introduced into the chamber. The data from APS bins are aggregated to match the size bins reported by PMS. For PMS bin 0.3 - 0.5 µm, APS bin < 0.523 µm was used for comparison.

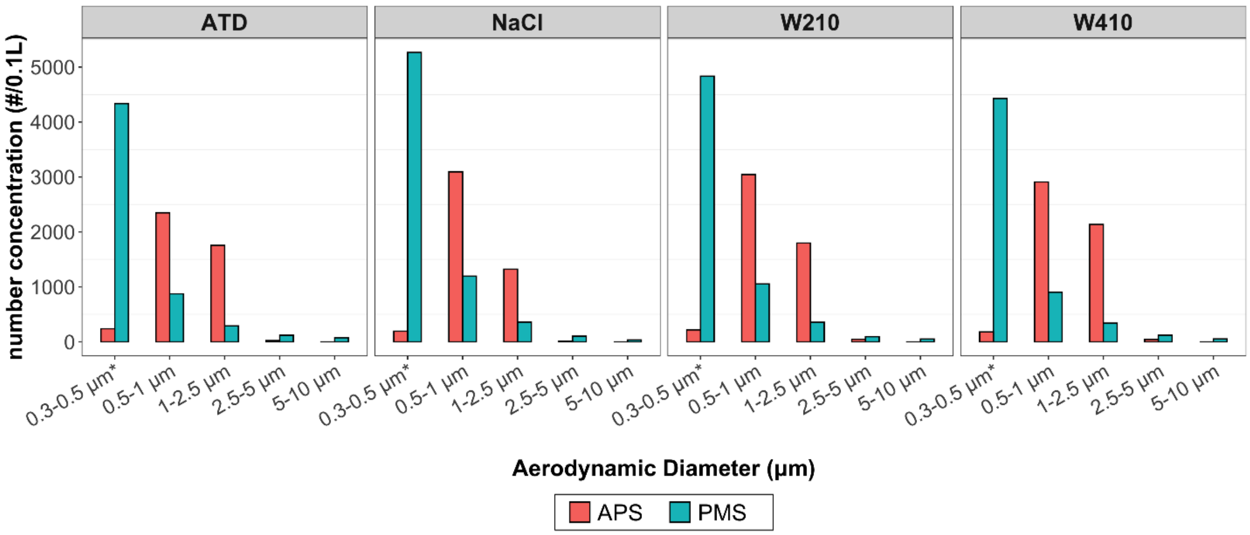

pp 14 - 15, lines 255 – 271 in the revised text:
"**Error! Reference source not found.** shows the typical particle size distributions reported by the PMS and the APS; the APS bins were aggregated to match the PMS. For all aerosols and all tested concentrations, the PMS appeared to underestimate particle counts for the size bin 0.5–1 µm and 1–2.5 µm. For larger size bins (2.5–5 µm and 5–10 µm), the PMS overestimate the particle counts. The particle count varies significantly in the lowest size bin (PMS: d_p= 0.3–0.5 µm; APS: d_p <0.523 µm). Both the APS and PMS use the optical channel. Since APS is a single particle instrument, its detection limit is based on the excitation wavelength, photodetector sensitivity, and particle optical properties; it is reported to be 0.37 µm. The PMS does not count single particle and relies on the internal calibration of the bulk scattering signal. The PMS OEM calibration significantly overestimates the counts in the 0.3–0.5 µm size range. For PMS, we measured the lower detection limit for the number concentration for the NaCl particles. In NaCl particle experiments, the initial (non-zero) response in the most sensitive PMS bin (d_p > 0.3 µm) was observed at the ~ 83 #/0.1 L as measured by APS. The PMS sensors seemed to follow the overall trends in the size distribution of the particles greater than 0.5 µm; however, the number concentration and particle sizing do not agree with the single-particle counter. Thus, calibration is needed if PMS is to be used for PM number concentration measurement."

3. In Table 2 (and all the rest of the paper), are aerodynamic size used for APS all the time? If so, why? Why not using optical size from the APS?

Reply: Yes, the aerodynamic size from the APS was used in Table 2 and the rest of the paper. Evaluating the correlation between the PMS measurements and APS aerodynamic size measurements is the focus of this study. Some previous studies have also demonstrated calibration based on the aerodynamic size from APS with PMS optical particle sensor [1-4]. The aerodynamic diameter provides better estimates for particle settling, inertial collection in the respiratory tract, which are essential for exposure assessment studies. We have updated the table caption and added discussion in the manuscript.

4. Figures in Response to Q2 by R2: Can you please add regression equations and R2? And the top right and bottom right figures have the same x and y axis but not the same data. Why?

Are these figures in the supplementary or main text? If not, it would be useful to add them

Reply: In our response to Q2 by Reviewer2, the top-right and bottom-right figures have the same plot title. This appeared to be an error. The figure has been corrected as follow:

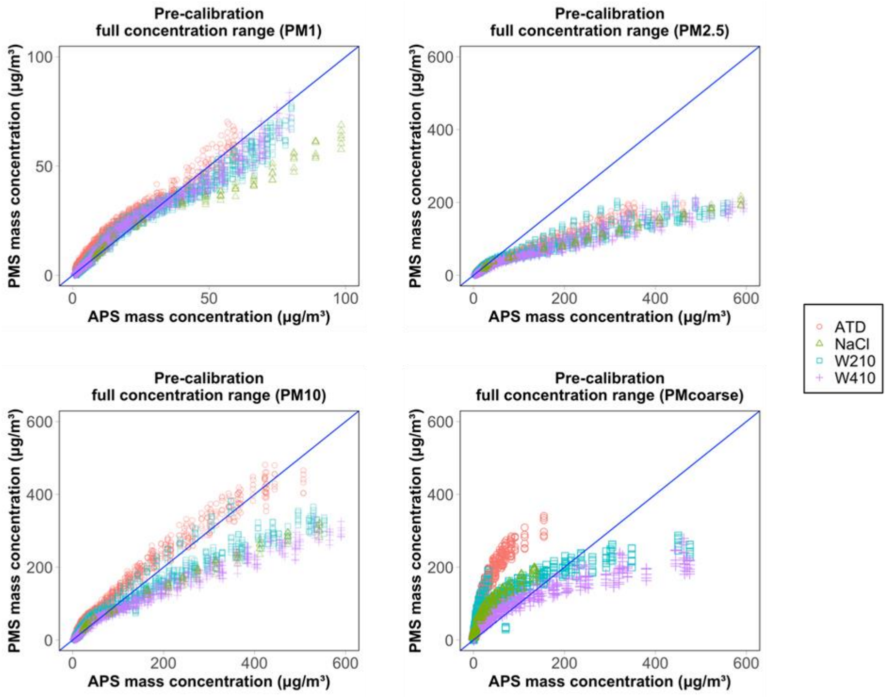

As this figure shows the pre-calibrated mass concentration metrics, the regression equations and R^2 cannot be calculated based on these data. We have updated the text describing this figure and included it in the supplementary information as S13 Fig. The text describing this figure in the manuscript has also been revised.

pp 19, lines 327 – 330 in the revised text:
"The poorer performance of the PMS for the coarse PM fraction was observed; the relationship between the estimated PMS and APS mass concentration values for the coarse size fraction (i.e., particle sizes from 2.5 to 10 µm) was markedly worse than smaller size fractions (S13 Fig)."

5. Line 31: please clarify "shifted" to larger or smaller size. Reading the results, I do not seem to see this "shift" clearly.

Reply: We've revised the text to clarify this point.

pp 2, lines 30 – 31 in the revised text:
"The results suggested particle size distribution using OEM calibration for PMS A003 sensor did not agree with the single particle sizer."

6. Final sentence in abstract: Please be specific of what are or is the "implications"? At the moment, it is vague and readers won't really know the implications. Is it PMS should not be used in high concentration environment? And what is considered high concentration

environment?

Reply: We've revised the text in the abstract to make the message clearer.

pp 2, lines 21 – 37 in the revised text:
"Low-cost optical scattering particulate matter (PM) sensors report total or size-specific particle counts and mass concentrations. The PM concentration and size are estimated by the original equipment manufacturers' (OEM) proprietary algorithms, which have inherent limitations since particle scattering depends on particles' properties such as size, shape, and complex index of refraction (CRI) as well as environmental parameters such as temperature and relative humidity (RH). As low-cost PM sensors are not able to resolve individual particles, there is a need to characterize and calibrate sensors' performance under a controlled environment. Here, we present improved calibration algorithms for Plantower PMS A003 sensor for mass indices and size-resolved number concentration. An aerosol chamber experimental protocol was used to evaluate sensor-to-sensor data reproducibility. The calibration was performed using four polydisperse test aerosols. The results suggested particle size distribution using OEM calibration for PMS A003 sensor did not agree with the single particle sizer. For the number concentration calibration, the linear model without adjusting for the aerosol properties and environmental conditions yields an absolute error (NMAE) of ~ 4.0% compared to the reference instrument. The calibration models adjusted for particle CRI and density account for non-linearity in the OEM's mass concentrations estimates with NMAE within 5.0%. The calibration algorithms developed in this study can be used in indoor air quality monitoring, occupational/industrial exposure assessments, or near-source monitoring scenarios where field calibration might be challenging."

7. Line 31 to 34 – If the error is small already without adjusting aerosol properties, then do we really need to care about making further adjustments? I am slightly confused with your overall message in this paper. On the one hand, you argued that accounting for CRI and density leads to an improvement in calibration; on the other hand, you argued that cautions should be given when making such adjustment. Since you also showed that no adjustment calibration is already very good, I wonder about what exactly do you want other researchers who are using PMS to do?

Reply: Thank you for pointing out the confusion. The models without accounting for particle properties already have low errors. Adding particle properties to the models does not significantly improve the models. Therefore, we decided to use the simpler models without introducing other variables. The sentence that caused confusion points out the limitation of our study, which is the calibration is only based on the particles within a narrow range of particle properties; therefore the caution should be given. For the clearance of the massage, we deleted the sentence.

8. Line 36-37: Can you clarify what does this mean "as the particle properties used in fitting were within a narrow range for challenge aerosols"?

Reply: In this study, four types of pre-selected polydisperse particles were used as the testing aerosols for calibration, including the Arizona Test Dust, 3M ceramic particles W210 and W410, and NaCl. These particles have CRIs ranging from 1.53 - 1.64 and density from 1.03 - 2.7 g/cm^3,

which were considered within a narrow range. Therefore, the generalizability of the calibration models developed in this study will be best when used in the settings where aerosols of exposures have CRIs and densities within this range. We have revised the text to make the message clearer.

pp 22, lines 370 – 373 in the revised text:
"We used particles in a relatively narrow range of CRIs (1.53 – 1.64) and densities (1.03 - 2.7 g/cm^3), and our RH was restricted within 17 - 80%. If the particle properties and environmental conditions of interest are significantly different from tested scenarios, one may need to consider these effects."

9. Line 37-38: Should this sentence be moved before its immediate previous sentence, i.e., to line 34?

Reply: Thank you for the suggestion. We have revised the text accordingly.

pp 2, lines 21 – 37 in the revised text:
"Low-cost optical scattering particulate matter (PM) sensors report total or size-specific particle counts and mass concentrations. The PM concentration and size are estimated by the original equipment manufacturers' (OEM) proprietary algorithms, which have inherent limitations since particle scattering depends on particles' properties such as size, shape, and complex index of refraction (CRI) as well as environmental parameters such as temperature and relative humidity (RH). As low-cost PM sensors are not able to resolve individual particles, there is a need to characterize and calibrate sensors' performance under a controlled environment. Here, we present improved calibration algorithms for Plantower PMS A003 sensor for mass indices and size-resolved number concentration. An aerosol chamber experimental protocol was used to evaluate sensor-to-sensor data reproducibility. The calibration was performed using four polydisperse test aerosols. The results suggested particle size distribution using OEM calibration for PMS A003 sensor did not agree with the single particle sizer. For the number concentration calibration, the linear model without adjusting for the aerosol properties and environmental conditions yields an absolute error (NMAE) of ~ 4.0% compared to the reference instrument. The calibration models adjusted for particle CRI and density account for non-linearity in the OEM's mass concentrations estimates with NMAE within 5.0%. The calibration algorithms developed in this study can be used in indoor air quality monitoring, occupational/industrial exposure assessments, or near-source monitoring scenarios where field calibration might be challenging."

10. Line 216 – explain briefly how the outliers were identified? What do "outside the measurement range of APS were removed"? I am not sure you can use this as a criterion to remove outliers.

Reply: Thank you for pointing this out. This sentence was meant to describe that removed the measurements outside the APS's measurement range. We have corrected the sentence to make this clearer. In the user manual of the APS, it was stated that the maximum recommended particle concentration is 1,000 $particles/cm^3$ at 0.5 µm with < 5% coincidence. We applied this limit to filter the data to avoid any coincidence error in the measurement.

pp 9, lines 162 – 165 in the revised text:
"Per APS specifications, the maximum recommended particle concentration is 1000 #/cm^3 at 0.5 µm with < 5% coincidence. Therefore, the total number concentration of the aerosols in the test chamber was maintained below 1000 #/cc (10^5 #/0.1L)."

p 11, lines 195 – 196 in the revised text:
"The collected data above the recommended level to avoid counting errors of APS (total particle count > 1000 #/ cm^3) was removed."

11. Line 283 – If the particle number is overestimated by so much (up to nearly 3000 times), then it is likely to be meaningless data, considering the huge contribution of large particles to particle mass. Can this be checked again?

Reply: We have double-checked the data. The PMS significantly miscategorized the particles smaller than 0.5 µm. We revised Figure 4 per the previous suggestion and added this point to the discussion. It is unclear if PMS uses the number concentration to calculate the mass concentration. The mass concentration shows less discrepancy between the PMS and the APS. We suspect the mass concentration was reported independently from the number concentration for PMS.

pp 14 - 15, lines 255 – 271 in the revised text:
"**Error! Reference source not found.** shows the typical particle size distributions reported by the PMS and the APS; the APS bins were aggregated to match the PMS. For all aerosols and all tested concentrations, the PMS appeared to underestimate particle counts for the size bin 0.5–1 µm and 1–2.5 µm. For larger size bins (2.5–5 µm and 5–10 µm), the PMS overestimate the particle counts. The particle count varies significantly in the lowest size bin (PMS: d_p =0.3–0.5 µm; APS: d_p <0.523 µm). Both the APS and PMS use the optical channel. Since APS is a single particle instrument, its detection limit is based on the excitation wavelength, photodetector sensitivity, and particle optical properties; it is reported to be 0.37 µm. The PMS does not count single particle and relies on the internal calibration of the bulk scattering signal. The PMS OEM calibration significantly overestimates the counts in the 0.3–0.5 µm size range. For PMS, we measured the lower detection limit for the number concentration for the NaCl particles. In NaCl particle experiments, the initial (non-zero) response in the most sensitive PMS bin (d_p > 0.3 µm) was observed at the ~ 83 #/0.1 L as measured by APS. The PMS sensors seemed to follow the overall trends in the size distribution of the particles greater than 0.5 µm; however, the number concentration and particle sizing do not agree with the single-particle counter. Thus, calibration is needed if PMS is to be used for PM number concentration measurement."

12. Figure 3: can you use a different Y-axis range for the bottom figures, e.g., upper limit of 1000?

Reply: The Y-axis range of Figure 3 has been revised.

Revised Figure 3:

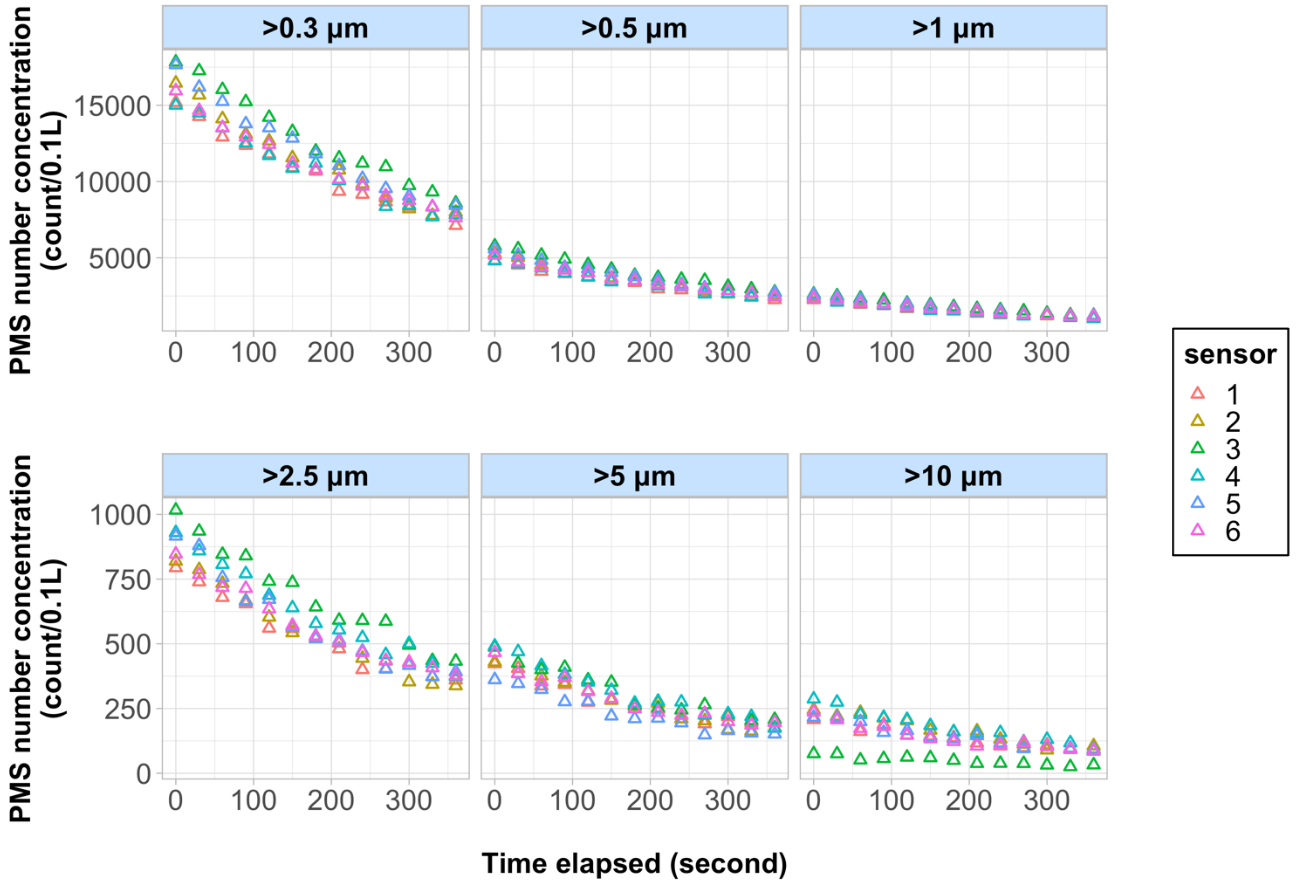

Fig 3. Time-series plots of the uncalibrated, 1-second number concentration measurement from the six PMS sensors during an experiment conducted under 30% relative humidity with W210 aerosols.

13. Line 286 – this is confusing, what does it mean when you say "reached 0 #/0.1L"?

Reply: Sorry for the confusion. It means when the measurement of PMS size bin >0.3 µm was/reached 0 #/0.1L, which also means that PMS detected no particles at all. We have made this clearer in the revised text.

pp 15 , lines 265 – 268 in the revised text:
"For PMS, we measured the lower detection limit for the number concentration for the NaCl particles. In NaCl particle experiments, the initial (non-zero) response in the most sensitive PMS bin (d_p > 0.3 µm) was observed at the ~ 83 #/0.1 L as measured by APS."

14. Line 287: why this means a lower detection limit for APS? In reality the size cut of APS is 370 nm but APS is 300 nm; also your Fig. 4 shows that the number (fraction) of particles <0.5 um is much larger from PPS than in APS.

Reply: Thank you for the comment, we revised the text as follow:

pp 15, lines 260 – 271 in the revised text:

"Fig 4 shows the typical particle size distributions reported by the PMS and the APS; the APS bins were aggregated to match the PMS. For all aerosols and all tested concentrations, the PMS appeared to underestimate particle counts for the size bin 0.5–1 µm and 1–2.5 µm. For larger size bins (2.5–5 µm and 5–10 µm), the PMS overestimate the particle counts. The particle count varies significantly in the lowest size bin (PMS: dp=0.3–0.5 µm; APS: dp <0.523 µm). Both the APS and PMS use the optical channel. Since APS is a single particle instrument, its detection limit is based on the excitation wavelength, photodetector sensitivity, and particle optical properties; it is reported to be 0.37 µm. The PMS does not count single particle and relies on the internal calibration of the bulk scattering signal. The PMS OEM calibration significantly overestimates the counts in the 0.3–0.5 µm size range. For PMS, we measured the lower detection limit for the number concentration for the NaCl particles. In NaCl particle experiments, the initial (non-zero) response in the most sensitive PMS bin (dp > 0.3 µm) was observed at the ~ 83 #/0.1 L as measured by APS. The PMS sensors seemed to follow the overall trends in the size distribution of the particles greater than 0.5 µm; however, the number concentration and particle sizing do not agree with the single-particle counter. Thus, calibration is needed if PMS is to be used for PM number concentration measurement."

15. Line 329: tended to be? those? Better?

Reply: We have corrected the wording to clarify this.

pp 17, lines 304 – 307 in the revised text:

"The BIC suggests that the models (shown in bold in S2 Table) with the adjustment for CRI and RH have similar NMAE. The table also includes the models based on the lower concentration data (data points with APS total number concentration <100 #/cm^3). The models based on the low PM concentration do not perform as well as the full range concentration models."

16. Fig. 6 – large uncertainty in the coarse particles particularly particles > 5 um could lead to the difficulty in calibrating particle mass? I wonder whether there is value to separate PM1, PM2.5 and PM10 in this figure (and similarly for Fig. 5)? The figures could be much smaller so that you can still show the information, as needed, but then it becomes clearer.

Reply: The mass concentration from the APS was estimated by adjusting for known particle densities in the APS software. We have confidence that the reference monitor could provide reliable mass concentration estimation for calibration since the densities of the standardized testing particles used in this study are known. We included the mass concentration metrics into a single figure because it allows a better comparison between different metrics. The same rationale applies to the figure for number concentration. We included figures that separate different bins of number concentration and PM1, PM2.5, and PM10 in the supplementary information Fig S13 and Fig S14, respectively.

Fig S14 in the supplementary information:

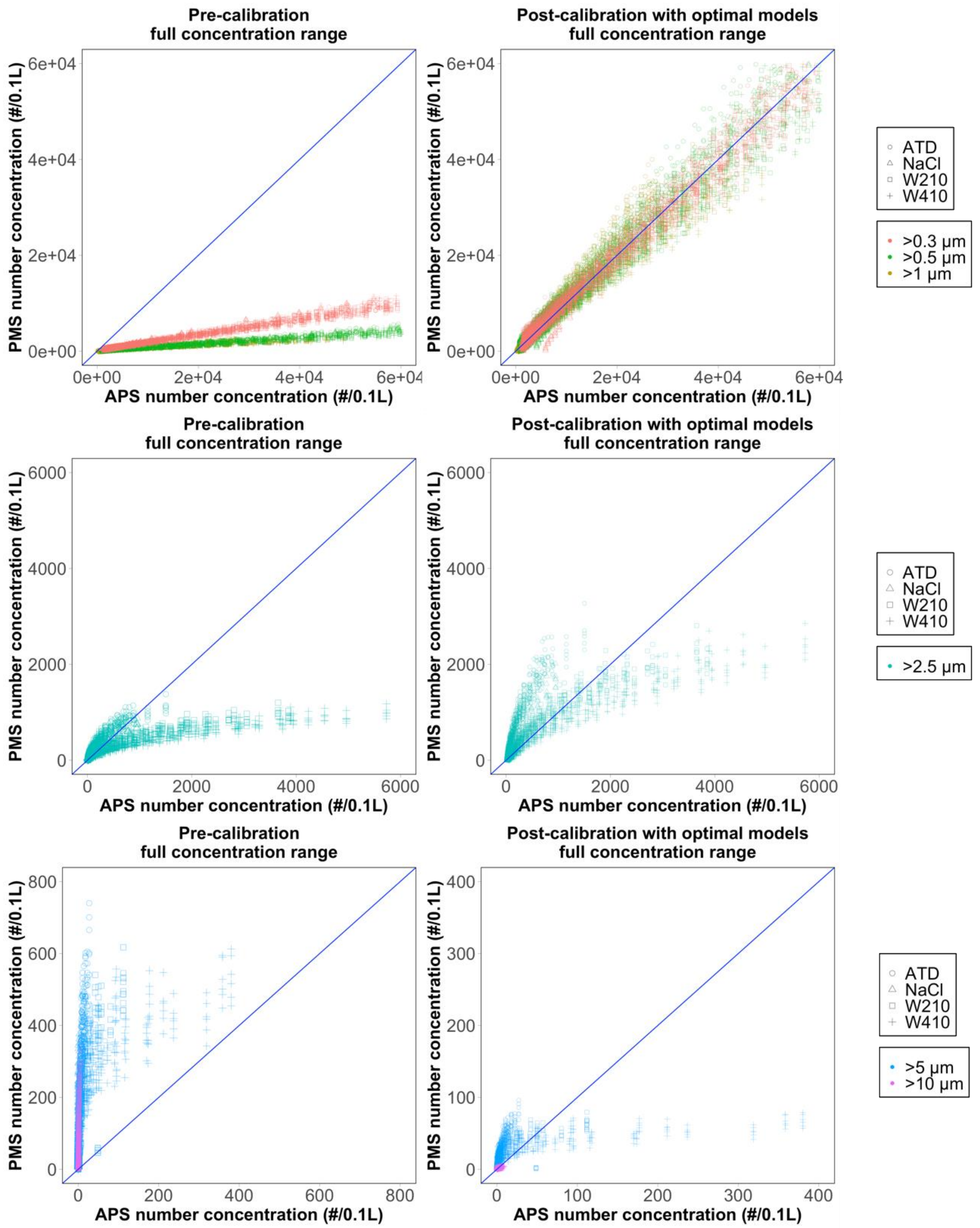

S14 Fig. A comparison of the pre-calibrated and post-calibrated number concentrations by different size bins. The blue line represents the 1:1 relationship between the PMS and APS concentration.

17. Reviewer 1 is concerned about the quality of the figures – e.g., figures are not very clear due to low resolution. I presume that when you submit the final version, you will submit higher resolution figures for production?

Reply: Well taken - We will upload high-resolution figures when submitting the final version.

References:

1. Levy Zamora M, Xiong F, Gentner D, Kerkez B, Kohrman-Glaser J, Koehler K. Field and Laboratory Evaluations of the Low-Cost Plantower Particulate Matter Sensor. Environ Sci Technol. 2019;53(2):838-49. Epub 2018/12/20. doi: 10.1021/acs.est.8b05174. PubMed PMID: 30563344.
2. Kuula J, Mäkelä T, Aurela M, Teinilä K, Varjonen S, González Ó, et al. Laboratory evaluation of particle-size selectivity of optical low-cost particulate matter sensors. Atmos Meas Tech. 2020;13(5):2413-23. doi: 10.5194/amt-13-2413-2020.
3. Tryner J, L'Orange C, Mehaffy J, Miller-Lionberg D, Hofstetter JC, Wilson A, et al. Laboratory evaluation of low-cost PurpleAir PM monitors and in-field correction using co-located portable filter samplers. Atmospheric Environment. 2020;220:117067. doi: https://doi.org/10.1016/j.atmosenv.2019.117067.
4. Kelly KE, Whitaker J, Petty A, Widmer C, Dybwad A, Sleeth D, et al. Ambient and laboratory evaluation of a low-cost particulate matter sensor. Environmental Pollution. 2017;221:491-500. doi: https://doi.org/10.1016/j.envpol.2016.12.039.

From previoussubmission

***PLOS One* Peer Review Comments and Responses**

July 6, 2021

We, the authors, would like to thank the Editor and all Reviewers for their thoughtful comments on how we can improve our manuscript. We reproduce each comment and provide responses below.

Comments from the Editor

Both reviewers found that this paper is interesting and of high value to researchers in the field. They raised a number of issues, primarily regarding the presentation and discussions on limitations.

Reply: Thanks. We have attempted to address all the issues raised by the reviewers. Please see the details below. All requested documents have been provided accordingly.

Comments from Reviewers

Reviewer #1

General comments: This study provides calibration algorithms for low-cost PMS A003 PM sensor as a function of particle size and concentration. The science behind the study looks fine and paper shows good interest for PLOS ONE readers. Overall, the results are promising and the manuscript is well written. However, the authors did not present detailed limitations of the method used in the study. I have some minor concerns before accepting the paper for publication.

Reply: Thank you. We have attempted to address the concerns.

Specific comments:
1. Minor edits are required throughout the text to improve clarity of the text when reading and to correct grammar.

Reply: We have made edits and corrected grammar to improve the clarity of the text.

pp 5, line 86 - 88 in the revised text:
"As low-cost sensors find applications in pollution monitoring, there is a need to characterize and calibrate their performance under various conditions, especially in controlled environments with standardized test aerosols."

pp 5 - 6, line 97 - 100 in the revised text:
"Third, calibrations based on short-term field colocations with reference instruments often depend on real-world conditions, and thus typically cannot be used to generalize wide-ranging concentrations and the effect of varying particle sources."

pp 14 - 15, line 261 - 262 in the revised text:
"…After identifying the optimal models using BIC and estimating model coefficients, the model was then applied to the pre-calibrated 1-minute PMS measurement to produce the post-calibrated concentrations for model evaluation."

pp 15, line 270 - 271 in the revised text:
"… where $C_{PMS\_post-cal}$ is the post-calibrated 1-minute averaged PMS concentration and C_{APS} is the 1-minute averaged APS concentration."

pp 16, line 287 - 288 in the revised text:
"The W410 mixture had slightly larger particles than W210 and had the same CRI as W210 [52]."

pp16, line 292 - 293 in the revised text:
"… For all aerosols and all tested concentrations, the PMS categorized some small fractions of particles into larger size bins (2.5–5 µm and 5–10 µm), which is not consistent with the…"

pp 17, line 306 - 307 in the revised text:
"…calibration based on a wider variety of aerosol types and environmental conditions is needed if PMS is to be used for PM concentration assessment."

pp 17, line 317 - 318 in the revised text:
"…and the APS reference number concentrations for different size ranges suggests that the matching size bins between the two instruments are well-correlated (S1 Fig)."

2. Aerosol Chamber Tests: what are the limitations?

Reply: In the revised text, we have extended the discussion of the limitations of aerosol chamber tests.

pp 23 - 24, line 429- 446 in the revised text:
"…In addition, environmental conditions related to real-world scenarios need to be considered when applying these models. We used particles in a relatively narrow range of CRIs, morphologies, and densities, and our RH was restricted within 20 - 80%. If the particle properties and environmental conditions of interest are significantly different from tested scenarios, one may need to consider these effects. Despite these limitations, this study has major implications, especially for using these sensors in high concentration environments, including indoor air quality monitoring, occupational/industrial exposure assessments, wildfire smoke, or near-source monitoring scenarios…"

3. Line 257: I think it should be equation 2.

Reply: Thanks for pointing this out. It has been corrected.

Equation 2 in the main text (p 15, line 268):

$$\text{NMAE}\ (\%) = \frac{\text{Mean}(|C_{\text{PMS\_post-cal}} - C_{\text{APS}}|)}{\text{Mean}(C_{\text{APS}})} \quad (2)$$

4. Table 4 shows summary of the calibration model for mass concentration. However the table is not explicitly described in the manuscript.

Reply: We have revised the text to describe Table 4 more explicitly.

pp 20, line 364 - 383 in the revised text:
"For fitting mass concentration data, an additional quadratic term was included, shown in Table 4 and S3 Table. As with the number concentration models, restricting the mass concentration model to only lower concentrations (data points with APS total number concentration <100 #/cm3) resulted in worse performance vs. model based on the entire concentration range. The optimal models (shown in bold in S3 Table) included quadratic terms of PMS measurement, terms related to particle properties and environmental conditions (CRI, density, and in some cases, RH) resulted in NMAE < 3.4% for full concentrations. Comparing to the relatively simple linear models without these added parameters (CRI, density, and RH terms), the improvements in NMAE for models adjusted for these parameters tended to be larger than those observed for the number concentration models. The inclusion of quadratic term of PMS measurement in these optimal models also highlighted the non-linearity in the OEM's mass concentrations estimates (Table 4 and S3 Table). In addition, the R2 values of the PM10 models (ranged between 0.85 – 0.88) are lower than the R2 values of the PM2.5 models (ranged between 0.94 – 0.96), which potentially indicated that the sensor performance dropped for particle size within the range of 2.5 – 10 um."

5. I suggest to add a separate section for study limitations.

Reply: Thanks for the suggestion. We extended the discussion of study limitation in the conclusion, as per comment 2:

pp 23 - 24, line 428- 445 in the revised text:
"…In addition, environmental conditions related to real-world scenarios need to be considered when applying these models. We used particles in a relatively narrow range of CRIs, morphologies, and densities, and our RH was restricted within 20 - 80%. If the particle properties and environmental conditions of interest are significantly different from tested scenarios, one may need to consider these effects. Despite these limitations, this study has major implications, especially for using these sensors in high concentration environments, including indoor air quality monitoring, occupational/industrial exposure assessments, wildfire smoke, or near-source monitoring scenarios.."

6. Conclusions section is well written but the authors should give a clear future direction.

Reply: We have revised the conclusions to make the future study direction clearer.

pp 23 - 24, line 423 - 451in the revised text:

"In this study, we evaluated the PMS sensors and reported the calibration algorithm for both number concentration and mass concentration. We found that the number concentrations can be corrected using a simple linear model, and mass concentrations can be better corrected using a polynomial model, although the BIC statistical considerations indicated models that including particle properties and environmental conditions are technically superior. When restricting the fit to the lower concentration, the accuracy of the model decreases for both number and mass concentration, and the larger size bins tended to have higher errors. In addition, environmental conditions related to real-world scenarios need to be considered when applying these models. We used particles in a relatively narrow range of CRIs, morphologies, and densities, and our RH was restricted within 20 - 80%. If the particle properties and environmental conditions of interest are significantly different from tested scenarios, one may need to consider these effects. Despite these limitations, this study has major implications, especially for using these sensors in high concentration environments, including indoor air quality monitoring, occupational/industrial exposure assessments, wildfire smoke, or near-source monitoring scenarios. Since the test aerosols used in this study are applicable for several occupational health scenarios, a better exposure assessment could be achieved. In the application of near-source and indoor air quality monitoring where field calibration might be challenging, these low-cost sensors along with the calibration algorithms developed within a higher concentration range applied could serve as a substitute for the traditional gravimetric instruments to provide accurate air quality data.

7. Figures quality also needs improvement.

Reply: We have updated the current Figure 5 and Figure 6 to improve their quality.

Revised Figure 5:

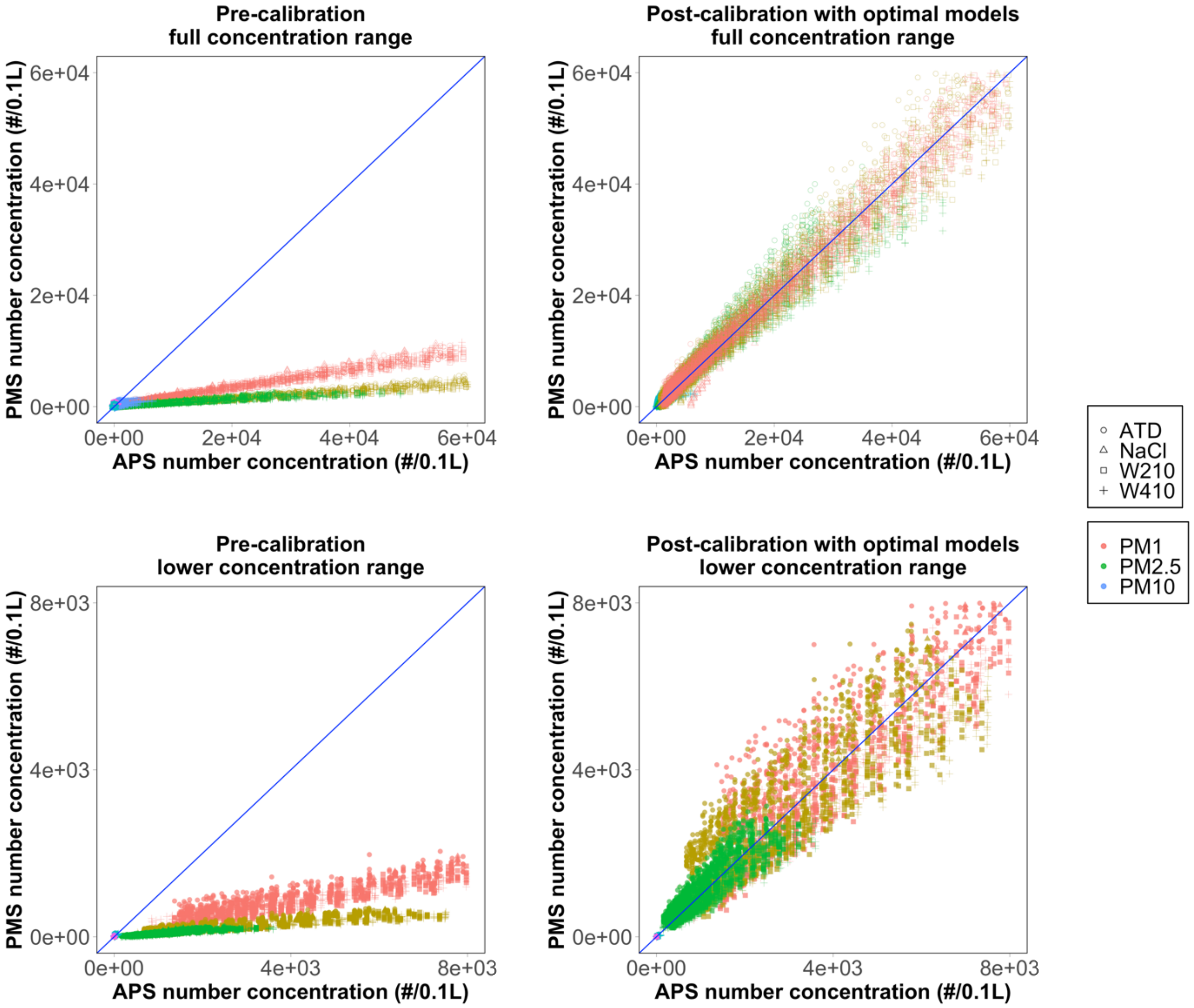

Fig 1. A comparison of the pre-calibrated and post-calibrated mass concentration by full and lower concentration range. The blue line represents the 1:1 relationship between the PMS and APS concentration.

Revised Figure 6:

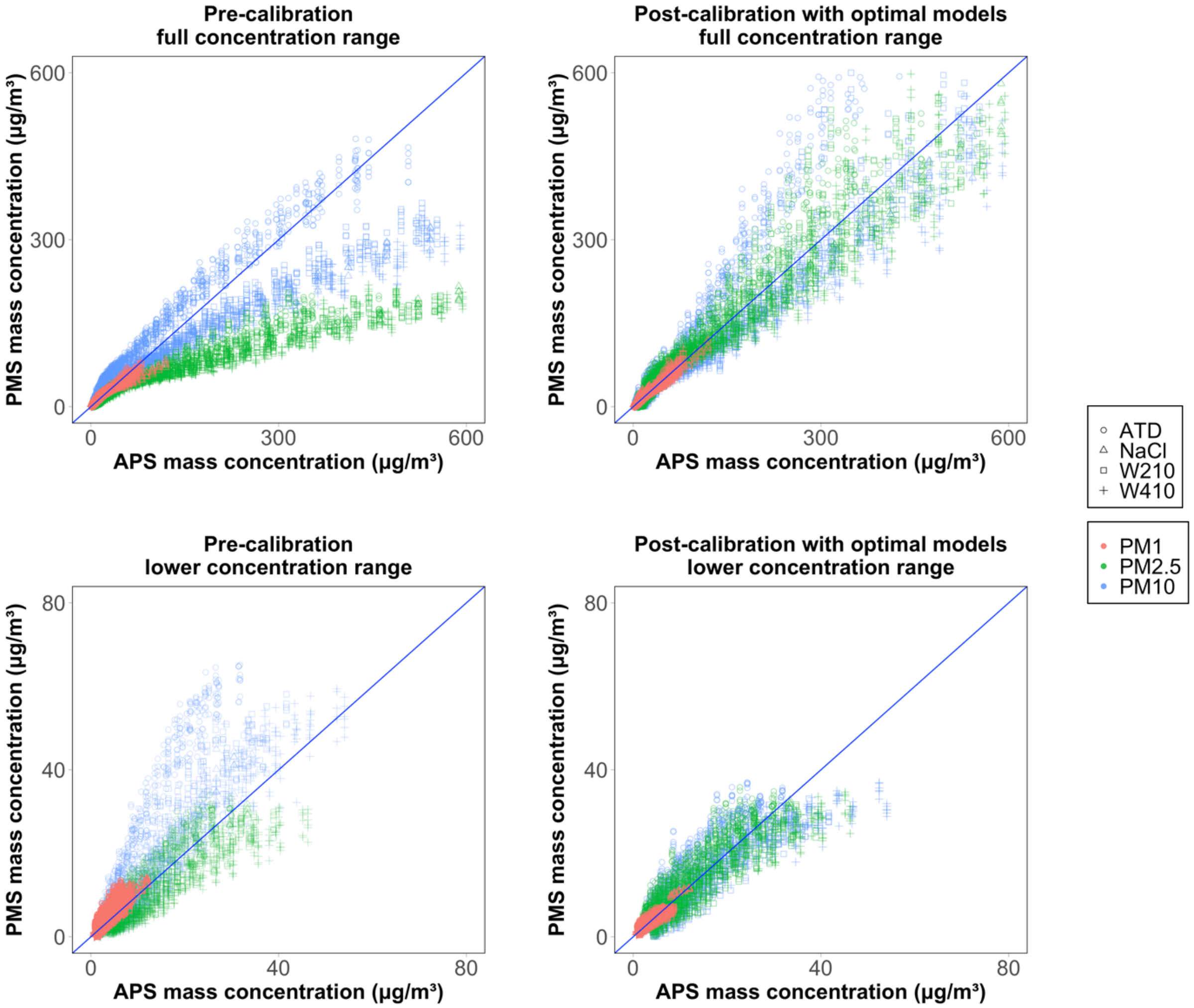

Fig 2. A comparison of the pre-calibrated and post-calibrated number concentration by full and lower concentration range. The blue line represents the 1:1 relationship between the PMS and APS concentration.

Reviewer #2:

General Comments: This article conducted a valuable laboratory analysis of a low-cost optical particle counter against the Aerodynamic Particle Sizer research instrument. While I have no concern regarding the laboratory experiments conducted, improvement is needed regarding how the data are analyzed, presented, and interpreted to the reader. These include the following:

1) The authors appear to discount the importance that there is a mismatch between the lowest channel of the APS and the lowest channel of the PMS. Given the highest number concentrations occur in this channel (e.g., their Figure 2 shows >0.3 counts ranging ~7000-15000 #/0.1L compared to far lower values for other channels), any differences in the upper and lower size limits of the two channels is an important discrepancy to highlight and take into account throughout the article. The question here is – what is the

actual lower detection limit of the APS? If, the PMS detects to a lower detection limit, then the relative differences in the dN/dlogDp size bin in Figure 3 is explainable.

Reply: Since APS is the reference instrument in this study, we cannot answer the lower detection limit of APS unless there is another reference instrument that we can compare with the APS measurement. To address the other part of this question, we conducted another set of tests which limited the APS total number concentration below 1 #/cm^3 with the same polydisperse aerosols (ATD, NaCl, w210, and w410), since in our previous chamber tests the lowest APS total number concentration reached were around 8 #/cm^3. Our new tests showed that, when the measurement of PMS bin > 0.3 µm reached 0 #/0.1L, the total number concentration measurement of APS was around 83 #/0.1L, which means that APS detects a lower detection limit. We have discussed this in the revised text.

pp 16 - 17, line 298 - 301 in the revised text:
"…The test also showed when the particle counts for the PMS size bin > 0.3 µm reached 0 #/0.1L, the total number concentration measurement of APS was around 83 #/0.1L, which meant that APS detected a lower detection limit. Overall, the…"

2) One outstanding question regarding the PMS sensor is their ability to detect the coarse particle size range (PM2.5-10). If the PM2.5 fraction is high, the PM10 results shown may not reveal whether the sensor performs well in high dust scenarios. The number count results indicate a performance drop for larger size channels. It is recommended that authors add an analysis of the PM2.5-10 fraction to the mass concentration analyses and discussion of the sensor performance for this size range. Given Figure 2 indicates the # counts in these larger size fractions may be very low, authors should indicate their level of confidence in larger particle size fraction data for both the number and mass-based analyses.

Reply: Thank you for the suggestion. In table 4 shows the results of the mass-based analyses, the R^2 values of the PM_{10} models (ranged between 0.85 – 0.88) are lower than the R^2 values of the $PM_{2.5}$ models (ranged between 0.94 – 0.96), which potentially indicated that the sensor performance dropped for particle size within the range of 2.5 – 10 um. Our way to quantify the level of confidence in larger particle size fraction data for both the number and mass-based analyses is based on the R^2 value and the normalized mean absolute error (NMAE) of the models evaluated. A model with a higher R^2 value and lower NMAE was considered a better fit than a model with a lower R^2 value and higher NAME. The R^2 values and NMAEs of the models are shown in Table 3 and Table 4 in the main text for number and mass-based analyses, respectively. This point has been addressed in the revised text.

pp 20 - 21, line 374 - 383 in the revised text:
"…In addition, the R^2 values of the PM_{10} models (ranged between 0.85 – 0.88) are lower than the R^2 values of the $PM_{2.5}$ models (ranged between 0.94 – 0.96), which potentially indicated that the sensor performance dropped for particle size within the range of 2.5 – 10 um."

3) Authors at the beginning and end of the manuscript state that scenarios such as wildfire smoke, near-source environments, and other high concentration environments necessitate this research. In the conclusions, they state "major implications." However, this seems to be a disconnect given the test aerosols chosen and that no true mass measurement was used as a reference. The interpretation of the

mass concentration results should be tempered and qualified that the APS has its own set of assumptions and is not a true mass measurement. In addition, the authors should describe how their test aerosols represent (or do not) some of the cited scenarios such as wildfire smoke, near-source environments (e.g., diesel emissions), and concentration ranges anticipated in those environments.

Reply: Thank you. In the conclusions, we have attempted to address these points.

pp 23 - 24, line 423 - 451 in the revised text:
"In this study, we evaluated the PMS sensors and reported the calibration algorithm for both number concentration and mass concentration. We found that the number concentrations can be corrected using a simple linear model, and mass concentrations can be better corrected using a polynomial model, although the BIC statistical considerations indicated models that including particle properties and environmental conditions are technically superior. When restricting the fit to the lower concentration, the accuracy of the model decreases for both number and mass concentration, and the larger size bins tended to have higher errors. In addition, environmental conditions related to real-world scenarios need to be considered when applying these models. We used particles in a relatively narrow range of CRIs, morphologies, and densities, and our RH was restricted within 20 - 80%. If the particle properties and environmental conditions of interest are significantly different from tested scenarios, one may need to consider these effects. Despite these limitations, this study has major implications, especially for using these sensors in high concentration environments, including indoor air quality monitoring, occupational/industrial exposure assessments, wildfire smoke, or near-source monitoring scenarios. Since the test aerosols used in this study are applicable for several occupational health scenarios, a better exposure assessment could be achieved. In the application of near-source and indoor air quality monitoring where field calibration might be challenging, these low-cost sensors along with the calibration algorithms developed within a higher concentration range applied could serve as a substitute for the traditional gravimetric instruments to provide accurate air quality data.

4) Data availability - the supplemental files do not contain any data sets, nor are there data sets in the body of the manuscript. This needs to be addressed to meet the journal requirements.

Reply: The dataset has been uploaded along with the main manuscript and the supplemental files to the submission system.

Specific comments:

Line 63-64: Add citation for the statement beginning "Estimates show…"

Reply: The text has been revised accordingly.

pp 4, line 63 - 65 in the revised text:
"…Estimates show that approximately 3% of cardiopulmonary and 5% of lung cancer deaths are attributed to exposures to $PM_{2.5}$ (particles less than 2.5 μm in diameter) globally [8]."

Line 69-70: Recommend revision for clarity – do authors mean the US EPA set the NAAQS under the Clean Air Act and therefore requires monitoring by states for these PM parameters?

Reply: Yes. It referred to the NAAQS set by the US EPA under the Clean Air Act. We have made this clearer in the revised text (pp XX, line 69 – 70).

pp 4, line 69 - 71 in the revised text:
"…As part of the Clean Air Act, the National Ambient Air Quality Standard (NAAQS) set by the U.S. Environmental Protection Agency (EPA) has adopted and established monitoring requirements for six criteria air pollutants, including $PM_{2.5}$ and PM_{10} [13, 14]."

Line 97: Recommend looking into "developing countries" is the best description. World Bank often uses "Low and Middle Income Countries" (LMICs)

Reply: Thank you for the suggestion. We understood that the use of "Low and Middle Income Countries" might be a more suitable word when refers to the economics of a country. In our cited references for this sentence, the term "developing countries" was used. Therefore, we think the use of "developing countries" is a more appropriate word in this context.

Line 97-100: Revise for clarity – do you mean "field colocations…cannot be conducted"?

Reply: Yes. This appeared to be a typo in our unrevised text. In the revised text, we have corrected it.

pp 6, line 103 - 105 in the revised text:
"…Also, in developing countries with less strict regulations, the PM level associated with, e.g., traffic emissions [34], agricultural waste burning [35], indoor cooking [36] is significantly higher, but where field colocations for calibration studies cannot be conducted."

Paragraph beginning on line 103: Given this experiment relies upon optical measurement methodology for both the sensor and the reference, it would be helpful to separate measurement principle from implementation of the principle in a sensor package. For example, line 105 seems to discount a measurement principle of bulk light scatter, which would include nephelometers that provide accurate measurements of the particle scattering coefficient however need calibration to estimate mass concentration.

Reply: We have revised the sentences to emphasize the limitation of the sensor that using bulk light scatter principle on particle mass concentration measurement.

pp 6, line 110 - 111 in the revised text:
"…Aerosol photometers that measure the bulk light scatter of multiple particles simultaneously have limited success in measuring mass concentration [30]."

Line 164: Can authors explain the "atmospheric" concentration channel and their choice to use the "factory" option? What correction factor is applied to the atmospheric condition channels and why was this not used, given the authors' stated motivation to inform use of these sensors in outdoor air quality measurement scenarios. How do the results for the factory channel apply to the other channel's measured

output? Given both data sets appear, this would be a valuable analysis to provide in supplemental information.

Reply: Thanks. According to the sensor manufacturer manual, the factory concentration channel reported mass concentration with a correction factor of 1 applied to the data, which means the number reported for this channel can be viewed as the "raw" data. However, for the atmospheric concentration channel, the manufacturer does not explicitly describe the correction factor and the algorithm applied to the data. The goal of this work is to calibrate the raw readings from the sensor. Therefore, we chose the factory channel as our primary interest for calibration. This is mentioned in the original text.

pp 9, line 168 - 171 in the revised text:

"…The mass concentrations are reported for two settings: "factory" and "atmospheric" conditions. The factory condition applies a correction factor (CF) of unity to the concentration measured, whereas the "atmospheric" condition is designed for use in the ambient environment."

Line 188: Add NaCl to the list.

Reply: The text has been revised accordingly.

pp 11, line 194 - 196 in the revised text:

"We tested four polydisperse aerosols: Arizona Test Dust (ATD) (Powder Technology Incorporated, Arden Hills, MN), polydisperse W210, and W410 ceramic particles (3M™, St. Paul, MN), and sodium chloride (NaCl) particles."

Line 314: What mass density was used for the APS mass concentration estimates? The "assumed" density on Table 1? Is the density used in Table 4's calibration model also the values in Table 1? Last, this sentence only specifies PM2.5 but other mass fractions are also analyzed.

Reply: The assumed density in Table 1 for each testing particle was used for the APS mass concentration estimates. The results in Table 4 are also based on the assumed density in Table 1. We have clarified this in the revised text. We have also updated this line along with Table 2 with the reference APS bins for calibrating PMS mass concentration.

pp 18, line 331 - 336 in the revised text:

"…Based on the same rationale, for mass concentrations, the corresponding APS mass concentration measurement was chosen as the reference for calibrating the PMS mass concentration index. For example, APS PM2.5 mass concentration was chosen as the reference (independent variable) for calibrating the PMS PM2.5 mass concentration (dependent variable). The assumed density for each testing aerosol in Table 1 was used to obtain the APS mass concentration measurement for calibration."

Table 4 in the revised text:

"**Table 4.** Summary of the calibration model for mass concentration [a], R^2, Bayesian information criterion (BIC), and the normalized mean absolute error (NMAE) of the calibration model.

Indices	Equation	Regression [b]	R^2	BIC	NMAE

Full concentration range (APS total number concentration between 0 – 1000 #/ cm^3) (n = 4,134)					
PM_1	Linear	y = 1.06 x	0.96	25852	3.11%
	Polynomial	y = 0.76 x + 0.007 x^2	0.97	24480	2.41%
	Linear + CRI + density	y = 1.13 x + 13.88 CRI - 10.13 density	0.97	24181	2.84%
	Polynomial + CRI + density	y = 0.83 x + 0.01x^2 + 14.44 CRI - 9.58 density	0.98	23432	2.33%
$PM_{2.5}$	Linear	y = 2.29 x	0.94	42435	4.53%
	Polynomial	y = 1.55 x + 0.006 x^2	0.96	41341	3.41%
	Linear + CRI + RH	y = 2.51 x - 23.27 CRI + 0.36 RH	0.95	41565	4.07%
	Polynomial + CRI + RH	y = 1.80 x + 0.004 x^2 - 15.55 CRI + 0.42 RH	0.96	41152	3.44%
PM_{10}	Linear	y = 1.53 x	0.85	49963	3.56%
	Polynomial	y = 0.72 x - 0.003 x^2	0.88	48959	2.61%
	Linear + CRI + RH	y = 1.69 x - 39.14 CRI + 0.56 RH	0.87	49544	3.31%
	Polynomial + CRI + RH	y = 0.73 x + 0.003 x^2 - 17.94 CRI + 0.75 RH	0.88	48931	2.61%
Lower concentration range (APS total number concentration < 100 #/ cm^3) (n = 1,838)					
PM_1	Linear	y = 0.72 x	0.90	6211	10.10%
	Polynomial	y = 0.91 x - 0.02 x^{2asn}	0.91	6053	9.23%
	Linear + CRI + density	y = 0.57 x + 4.80 CRI - 2.68 density	0.93	5746	8.16%
	Polynomial + CRI + density	y = 0.80 x - 0.02 x^2 + 4.93 CRI - 2.95 density	0.93	5694	8.08%
$PM_{2.5}$	Linear	y = 1.10 x	0.91	11170	9.14%
	Polynomial	y = 1.34 x - 0.01 x^2	0.91	11087	8.80%
	Linear + CRI + RH	y = 0.97 x -1.87 CRI + 0.16 RH	0.92	10890	8.01%
	Polynomial + CRI + RH	y = 1.14 x - 0.006 x^2 - 2.43 CRI + 0.17 RH	0.92	10885	7.97%
PM_{10}	Linear	y = 0.63 x	0.89	11878	9.30%
	Polynomial	y = 0.86 x - 0.01 x^2	0.90	11686	8.53%
	Linear + CRI + RH	y = 0.54 x - 2.15 CRI + 0.20 RH	0.91	11627	8.01%
	Polynomial + CRI + RH	y = 0.78 x - 0.004 x^2 - 3.57 CRI + 0.21 RH	0.91	11461	7.75%

[a] The APS mass concentration measurement was obtained with the assumed density for testing aerosols in Table 1.

[b] y: APS measurement; x: PMS measurement.

Definition of abbreviations: n = number of datapoints; CRI = complex index of refraction; RH = relative humidity; BIC = Bayesian information criteria; NMAE = normalized mean absolute error."

Table 2 in the revised text:

"**Table 1.** The PMS manufacturer's specified size bins and mass indices with the corresponding reference APS size bins for calibration.

**PMS indices**	**Reference APS indices**
Number concentration	
> 0.3 µm	counts aggregated from all size bins (< 0.523 µm and 0.542 – 19.81 µm)
> 0.5 µm	counts aggregated from size bins 0.542 – 19.81 µm

> 1 µm	counts aggregated from size bins 1.037 – 19.81 µm
> 2.5 µm	counts aggregated from size bins 2.642 – 19.81 µm
> 5 µm	counts aggregated from size bins 5.048 – 19.81 µm
> 10 µm	counts aggregated from size bins 10.37 – 19.81 µm
Mass concentration	
PM1	mass aggregated from size bin < 0.523 µm – 0.965 µm
PM2.5	mass aggregated from size bin < 0.523 µm – 2.458 µm
PM10	mass aggregated from size bin < 0.523 µm – 9.647 µm

"

Line 319: Recommend incorporating into the abstract / conclusions the significant model performance decrease for larger size particles. As noted earlier, recommend PM2.5-10 mass concentration be added as an analysis which will confirm if that size fraction is poorly measured by the sensor.

Reply: Thank you. We have rewritten the conclusions accordingly.

pp 23 - 24, line 423 - 451 in the revised text:

"In this study, we evaluated the PMS sensors and reported the calibration algorithm for both number concentration and mass concentration. We found that the number concentrations can be corrected using a simple linear model, and mass concentrations can be better corrected using a polynomial model, although the BIC statistical considerations indicated models that including particle properties and environmental conditions are technically superior. When restricting the fit to the lower concentration, the accuracy of the model decreases for both number and mass concentration, and the larger size bins tended to have higher errors. In addition, environmental conditions related to real-world scenarios need to be considered when applying these models. We used particles in a relatively narrow range of CRIs, morphologies, and densities, and our RH was restricted within 20 - 80%. If the particle properties and environmental conditions of interest are significantly different from tested scenarios, one may need to consider these effects. Despite these limitations, this study has major implications, especially for using these sensors in high concentration environments, including indoor air quality monitoring, occupational/industrial exposure assessments, wildfire smoke, or near-source monitoring scenarios. Since the test aerosols used in this study are applicable for several occupational health scenarios, a better exposure assessment could be achieved. In the application of near-source and indoor air quality monitoring where field calibration might be challenging, these low-cost sensors along with the calibration algorithms developed within a higher concentration range applied could serve as a substitute for the traditional gravimetric instruments to provide accurate air quality data.

Figure 6: The PM1 results are barely viewable, PM2.5 partially blocked. Perhaps reorder the parameters for better viewing.

Reply: Figure 6 has been updated for better viewing of the PM_1 results.

Revised Figure 6:

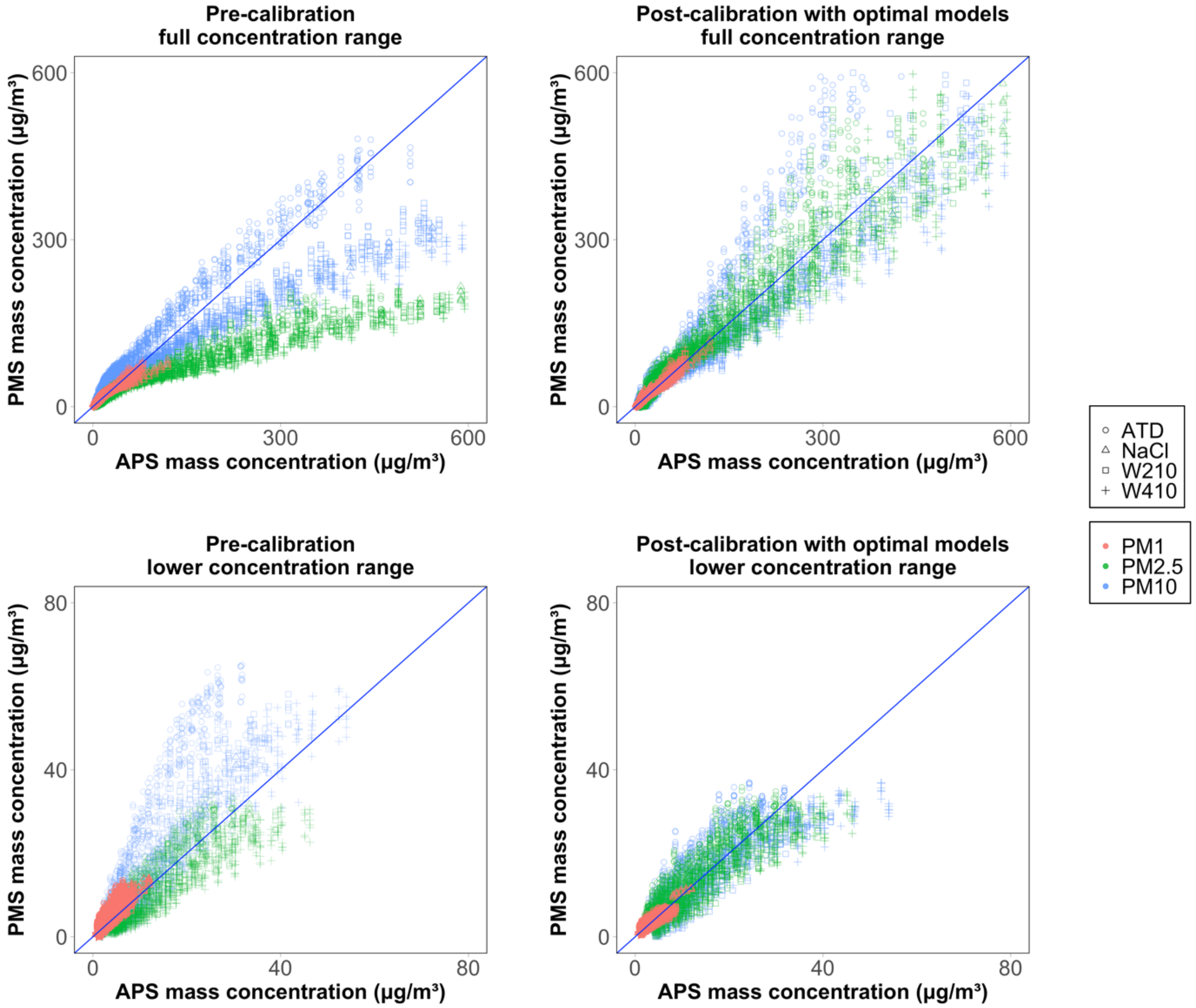

Fig 3. A comparison of the pre-calibrated and post-calibrated number concentration by full and lower concentration range. The blue line represents the 1:1 relationship between the PMS and APS concentration.
