# Supplementary material for: Assessing the value of complex refractive index and particle density for calibration of low-cost particle matter sensor for size-resolved particle count and PM2.5 measurements
Source: PLoS One. 2021 Nov 11;16(11):e0259745. doi: 10.1371/journal.pone.0259745 (PMC8584671; doi:10.1371/journal.pone.0259745)
Supplement: Attachment — Submitted filename: Response to Reviewers.docx [file pone.0259745.s018.docx]

***PLOS One* Peer Review Comments and Responses**

July 30, 2021

We, the authors, would like to thank the Editor and all Reviewers for their thoughtful comments on how we can improve our manuscript. We reproduce each comment and provide responses below.

# Comments from the Editor

Thank you for submitting your manuscript to PLOS ONE. After careful consideration, we feel that it has merit but does not fully meet PLOS ONE’s publication criteria as it currently stands. Therefore, we invite you to submit a revised version of the manuscript that addresses the points raised during the review process.

Please carefully review the comments by both reviewers. When revising this manuscript, please take particular attention to the presentation of results and the discussions on the limitations / uncertainties.

Please submit your revised manuscript by 3 Aug 2021. If you will need more time than this to complete your revisions, please reply to this message or contact the journal office at plosone@plos.org. When you're ready to submit your revision, log on to https://www.editorialmanager.com/pone/ and select the 'Submissions Needing Revision' folder to locate your manuscript file.

Please include the following items when submitting your revised manuscript:

- A rebuttal letter that responds to each point raised by the academic editor and reviewer(s). You should upload this letter as a separate file labeled 'Response to Reviewers'.
- A marked-up copy of your manuscript that highlights changes made to the original version. You should upload this as a separate file labeled 'Revised Manuscript with Track Changes'.
- An unmarked version of your revised paper without tracked changes. You should upload this as a separate file labeled 'Manuscript'.

If you would like to make changes to your financial disclosure, please include your updated statement in your cover letter. Guidelines for resubmitting your figure files are available below the reviewer comments at the end of this letter.

**Additional Editor Comments**

Both reviewers found that this paper is interesting and of high value to researchers in the field. They raised a number of issues, primarily regarding the presentation and discussions on limitations.

**Reply**: Thanks. We have attempted to address all the issues raised by the reviewers. Please see the details below. All requested documents have been provided accordingly.

# Comments from Reviewers

**Reviewer #1**

General comments: This study provides calibration algorithms for low-cost PMS A003 PM sensor as a function of particle size and concentration. The science behind the study looks fine and paper shows good interest for PLOS ONE readers. Overall, the results are promising and the manuscript is well written. However, the authors did not present detailed limitations of the method used in the study. I have some minor concerns before accepting the paper for publication.

**Reply**: Thank you. We have attempted to address the concerns.

Specific comments:
1. Minor edits are required throughout the text to improve clarity of the text when reading and to correct grammar.

**Reply**: We have made edits and corrected grammar to improve the clarity of the text.

**pp 5, line 83 - 85 in the revised text:**

“As low-cost sensors find applications in pollution monitoring, there is a need to characterize and calibrate their performance under various conditions, especially in controlled environments with standardized test aerosols.”

**pp 5 - 6, line 93 - 96 in the revised text:**

“…Third, calibrations based on short-term field colocations with reference instruments often depend on real-world conditions, and thus typically cannot be used to generalize wide-ranging concentrations and the effect of varying particle sources.”

**pp 14 - 15, line 251 - 253 in the revised text:**

“After identifying the optimal models using BIC and estimating model coefficients, the model was then applied to the pre-calibrated 1-minute PMS measurement to produce the post-calibrated concentrations for model evaluation.”

**pp 15, line 258 - 259 in the revised text:**

“… where $C_{PMS\_post-cal}$ is the post-calibrated 1-minute averaged PMS concentration and $C_{APS}$ is the 1-minute averaged APS concentration.”

**pp 16, line 274 - 275 in the revised text:**

“The W410 mixture had slightly larger particles than W210 and had the same CRI as W210 [52].”

**pp 16, line 279 - 280 in the revised text:**

“… For all aerosols and all tested concentrations, the PMS categorized some small fractions of particles into larger size bins (2.5–5 µm and 5–10 µm), which is not consistent with the…”

**pp 16 - 17, line 289 - 291 in the revised text:**

“…calibration based on a wider variety of aerosol types and environmental conditions is needed if PMS is to be used for PM concentration assessment.”

**pp 17, line 300 - 302 in the revised text:**

“…and the APS reference number concentrations for different size ranges suggests that the matching size bins between the two instruments are well-correlated (S1 Fig).”

2. Aerosol Chamber Tests: what are the limitations?

**Reply**: In the revised text, we have extended the discussion of the limitations of aerosol chamber tests.

**pp 24, line 397- 404 in the revised text:**

“…In addition, environmental conditions related to real-world scenarios need to be considered when applying these models. We used particles in a relatively narrow range of CRIs, morphologies, and densities, and our RH was restricted within 20 - 80%. If the particle properties and environmental conditions of interest are significantly different from tested scenarios, one may need to consider these effects. Despite these limitations, this study has major implications, especially for using these sensors in high concentration environments, including indoor air quality monitoring, occupational/industrial exposure assessments, wildfire smoke, or near-source monitoring scenarios…”

3. Line 257: I think it should be equation 2.

**Reply**: Thanks for pointing this out. It has been corrected.

**Equation 2 in the revised text (pp 15, line 256):**

$NMAE (\%)=\frac{Mean(\left| C_{PMS\_post-cal}-C_{\mathrm{APS}} \right|)}{Mean(C_{\mathrm{APS}})}$	(2)

4. Table 4 shows summary of the calibration model for mass concentration. However the table is not explicitly described in the manuscript.

**Reply**: We have revised the text to describe Table 4 more explicitly.

**pp 20 - 21, line 340 - 356 in the revised text:**

“For fitting mass concentration data, an additional quadratic term was included, shown in Table 4 and S3 Table. As with the number concentration models, restricting the mass concentration model to only lower concentrations (data points with APS total number concentration <100 #/cm3) resulted in worse performance vs. model based on the entire concentration range. The optimal models (shown in bold in S3 Table) included quadratic terms of PMS measurement, terms related to particle properties and environmental conditions (CRI, density, and in some cases, RH) resulted in NMAE < 3.4% for full concentrations. Comparing to the relatively simple linear models without these added parameters (CRI, density, and RH terms), the improvements in NMAE for models adjusted for these parameters tended to be larger than those observed for the number concentration models. The inclusion of quadratic term of PMS measurement in these optimal models also highlighted the non-linearity in the OEM’s mass concentrations estimates (Table 4 and S3 Table). In addition, the R^2^ values of the PM_10_ models (ranged between 0.85 – 0.88) are lower than the R^2^ values of the PM_2.5_ models (ranged between 0.94 – 0.96), which potentially indicated that the sensor performance dropped for particle size within the range of 2.5 – 10 µm. The poorer performance for the larger size fraction was also confirmed by observing that the relationship between the PMS and reference APS mass concentration values for PM_coarse_ size fraction (i.e., particle sizes from 2.5 to 10 µm) was markedly worse than smaller size fractions.”

5. I suggest to add a separate section for study limitations.

**Reply**: Thanks for the suggestion. We have extended the discussion of study limitation in the conclusion, as per comment 2:

**pp 24, line 397- 404 in the revised text:**

“…In addition, environmental conditions related to real-world scenarios need to be considered when applying these models. We used particles in a relatively narrow range of CRIs, morphologies, and densities, and our RH was restricted within 20 - 80%. If the particle properties and environmental conditions of interest are significantly different from tested scenarios, one may need to consider these effects. Despite these limitations, this study has major implications, especially for using these sensors in high concentration environments, including indoor air quality monitoring, occupational/industrial exposure assessments, wildfire smoke, or near-source monitoring scenarios…”

6. Conclusions section is well written but the authors should give a clear future direction.

**Reply**: We have revised the conclusions to make the future study direction clearer.

**pp 23 - 24, line 391 - 408 in the revised text:**

“In this study, we evaluated the PMS sensors and reported the calibration algorithm for both number concentration and mass concentration. We found that the number concentrations can be corrected using a simple linear model, and mass concentrations can be better corrected using a polynomial model, although the BIC statistical considerations indicated models that including particle properties and environmental conditions are technically superior. When restricting the fit to the lower concentration, the accuracy of the model decreases for both number and mass concentration, and the larger size bins tended to have higher errors. In addition, environmental conditions related to real-world scenarios need to be considered when applying these models. We used particles in a relatively narrow range of CRIs, morphologies, and densities, and our RH was restricted within 20 - 80%. If the particle properties and environmental conditions of interest are significantly different from tested scenarios, one may need to consider these effects. Despite these limitations, this study has major implications, especially for using these sensors in high concentration environments, including indoor air quality monitoring, occupational/industrial exposure assessments, wildfire smoke, or near-source monitoring scenarios. Since the test aerosols used in this study are applicable for several occupational health scenarios, a better exposure assessment could be achieved. In the application of near-source and indoor air quality monitoring where field calibration might be challenging, these low-cost sensors along with the calibration algorithms developed within a higher concentration range applied could serve as a substitute for the traditional gravimetric instruments to provide accurate air quality data.”

7. Figures quality also needs improvement.

**Reply**: We have updated Figure 5 and Figure 6 to improve their quality.

**Revised Figure 5:**

**Fig 5. A comparison of the pre-calibrated and post-calibrated mass concentration by full and lower concentration range.** The blue line represents the 1:1 relationship between the PMS and APS concentration.

**Revised Figure 6:**

**Fig 6. A comparison of the pre-calibrated and post-calibrated number concentration by full and lower concentration range.** The blue line represents the 1:1 relationship between the PMS and APS concentration.

**Reviewer #2:**

General Comments: This article conducted a valuable laboratory analysis of a low-cost optical particle counter against the Aerodynamic Particle Sizer research instrument. While I have no concern regarding the laboratory experiments conducted, improvement is needed regarding how the data are analyzed, presented, and interpreted to the reader. These include the following:

1) The authors appear to discount the importance that there is a mismatch between the lowest channel of the APS and the lowest channel of the PMS. Given the highest number concentrations occur in this channel (e.g., their Figure 2 shows >0.3 counts ranging ~7000-15000 #/0.1L compared to far lower values for other channels), any differences in the upper and lower size limits of the two channels is an important discrepancy to highlight and take into account throughout the article. The question here is – what is the actual lower detection limit of the APS? If, the PMS detects to a lower detection limit, then the relative differences in the dN/dlogDp size bin in Figure 3 is explainable.

**Reply**: Since APS is the reference instrument in this study, we cannot answer the lower detection limit of APS unless there is another reference instrument that we can compare with the APS measurement. To address the other part of this question, we conducted another set of tests which limited the APS total number concentration below 1 #/cm^3^ with the same polydisperse aerosols (ATD, NaCl, W210, and W410), since in our previous chamber tests the lowest APS total number concentration reached were around 8 #/cm^3^. Our new tests showed that, when the measurement of PMS bin > 0.3 μm reached 0 #/0.1L, the total number concentration measurement of APS was around 83 #/0.1L, which means that APS detects a lower detection limit. We have discussed this in the revised text.

**pp 16, line 285 - 387 in the revised text:**

**“**…The test also showed when the particle counts for the PMS size bin > 0.3 µm reached 0 #/0.1L, the total number concentration measurement of APS was around 83 #/0.1L, which meant that APS detected a lower detection limit. Overall, the…”

2) One outstanding question regarding the PMS sensor is their ability to detect the coarse particle size range (PM2.5-10). If the PM2.5 fraction is high, the PM10 results shown may not reveal whether the sensor performs well in high dust scenarios. The number count results indicate a performance drop for larger size channels. It is recommended that authors add an analysis of the PM2.5-10 fraction to the mass concentration analyses and discussion of the sensor performance for this size range. Given Figure 2 indicates the # counts in these larger size fractions may be very low, authors should indicate their level of confidence in larger particle size fraction data for both the number and mass-based analyses.

**Reply**: Thank you for the suggestion. Table 4 shows the results of the mass-based analyses, the R^2^ values of the PM_10_ models (ranged between 0.85 – 0.88), which are lower than the R^2^ values of the PM_2.5_ models (ranged between 0.94 – 0.96), indicating that the sensor performance dropped with the inclusion of particle size within the range of 2.5 – 10 µm. Our way to quantify the level of confidence in larger particle size fraction data for both the number and mass-based analyses is based on the R^2^ value and the normalized mean absolute error (NMAE) of the models evaluated. A model with a higher R^2^ value and lower NMAE was considered a better fit than a model with a lower R^2^ value and higher NMAE. The R^2^ values and NMAEs of the models are shown in Table 3 and Table 4 in the main text for number and mass-based analyses, respectively.

Also, as you’ve requested, we conducted an additional analysis for PMcoarse. While the particle size distributions of the test aerosols generally had fewer PMcoarse particles than those in the PM_2.5_ size range, the relationship between the PMS sensor mass concentrations and reference APS mass concentractions are clearly worse for this size fraction compared to PM_2.5_ (figure below). PMcoarse is shown in the bottom right panel, in comparison to the relationships for PM_1_, PM_2.5_, and PM_10_ size fractions.

We have revised the text accordingly to reflect these points.

**pp 20 - 21, line 350 - 356 in the revised text:**

“…In addition, the R^2^ values of the PM_10_ models (ranged between 0.85 – 0.88) are lower than the R^2^ values of the PM_2.5_ models (ranged between 0.94 – 0.96), which potentially indicated that the sensor performance dropped for particle size within the range of 2.5 – 10 µm. The poorer performance for the larger size fraction was also confirmed by observing that the relationship between the PMS and reference APS mass concentration values for PMcoarse size fraction (i.e., particle sizes from 2.5 to 10 µm) was markedly worse than smaller size fractions.”

3) Authors at the beginning and end of the manuscript state that scenarios such as wildfire smoke, near-source environments, and other high concentration environments necessitate this research. In the conclusions, they state “major implications.” However, this seems to be a disconnect given the test aerosols chosen and that no true mass measurement was used as a reference. The interpretation of the mass concentration results should be tempered and qualified that the APS has its own set of assumptions and is not a true mass measurement. In addition, the authors should describe how their test aerosols represent (or do not) some of the cited scenarios such as wildfire smoke, near-source environments (e.g., diesel emissions), and concentration ranges anticipated in those environments.

**Reply**: Thank you. In the conclusions, we have attempted to address these points.

**pp 23 - 24, line 391 - 408 in the revised text:**

“In this study, we evaluated the PMS sensors and reported the calibration algorithm for both number concentration and mass concentration. We found that the number concentrations can be corrected using a simple linear model, and mass concentrations can be better corrected using a polynomial model, although the BIC statistical considerations indicated models that including particle properties and environmental conditions are technically superior. When restricting the fit to the lower concentration, the accuracy of the model decreases for both number and mass concentration, and the larger size bins tended to have higher errors. In addition, environmental conditions related to real-world scenarios need to be considered when applying these models. We used particles in a relatively narrow range of CRIs, morphologies, and densities, and our RH was restricted within 20 - 80%. If the particle properties and environmental conditions of interest are significantly different from tested scenarios, one may need to consider these effects. Despite these limitations, this study has major implications, especially for using these sensors in high concentration environments, including indoor air quality monitoring, occupational/industrial exposure assessments, wildfire smoke, or near-source monitoring scenarios. Since the test aerosols used in this study are applicable for several occupational health scenarios, a better exposure assessment could be achieved. In the application of near-source and indoor air quality monitoring where field calibration might be challenging, these low-cost sensors along with the calibration algorithms developed within a higher concentration range applied could serve as a substitute for the traditional gravimetric instruments to provide accurate air quality data.”

4) Data availability - the supplemental files do not contain any data sets, nor are there data sets in the body of the manuscript. This needs to be addressed to meet the journal requirements.

**Reply**: The dataset has been uploaded along with the manuscript and the supplemental files to the submission system.
 **Specific comments:**

Line 63-64: Add citation for the statement beginning “Estimates show…”

**Reply**: The text has been revised accordingly.

**pp 4, line 63 - 64 in the revised text:**

“…Estimates show that approximately 3% of cardiopulmonary and 5% of lung cancer deaths are attributed to exposures to PM_2.5_ (particles less than 2.5 μm in diameter) globally [8].”

Line 69-70: Recommend revision for clarity – do authors mean the US EPA set the NAAQS under the Clean Air Act and therefore requires monitoring by states for these PM parameters?

**Reply**: Yes. It referred to the NAAQS set by the US EPA under the Clean Air Act. We have made this clearer in the revised text (pp 4, line 68 - 71).

**pp 4, line 68 - 71 in the revised text:**

“…As part of the Clean Air Act, the National Ambient Air Quality Standard (NAAQS) set by the U.S. Environmental Protection Agency (EPA) has adopted and established monitoring requirements for six criteria air pollutants, including PM_2.5_ and PM_10_ [13, 14].”

Line 97: Recommend looking into “developing countries” is the best description. World Bank often uses “Low and Middle Income Countries” (LMICs)

**Reply**: Thank you for the suggestion. We understood that the use of “Low and Middle Income Countries” might be a more suitable word when refers to the economics of a country. In our cited references for this sentence, the term “developing countries” was used. Therefore, we think the use of “developing countries” is a more appropriate word in this context.

Line 97-100: Revise for clarity – do you mean “field colocations…cannot be conducted”?

**Reply**: Yes. This appeared to be a typo in our unrevised text. In the revised text, we have corrected it.

**pp 6, line 99 - 101 in the revised text:**

“…Also, in developing countries with less strict regulations, the PM level associated with, e.g., traffic emissions [34], agricultural waste burning [35], indoor cooking [36] is significantly higher, but where field colocations for calibration studies cannot be conducted.”

Paragraph beginning on line 103: Given this experiment relies upon optical measurement methodology for both the sensor and the reference, it would be helpful to separate measurement principle from implementation of the principle in a sensor package. For example, line 105 seems to discount a measurement principle of bulk light scatter, which would include nephelometers that provide accurate measurements of the particle scattering coefficient however need calibration to estimate mass concentration.

**Reply**: We have revised the sentences to emphasize the limitation of the sensor that using bulk light scatter principle on particle mass concentration measurement.

**pp 6, line 106 - 107 in the revised text:**

“…Aerosol photometers that measure the bulk light scatter of multiple particles simultaneously have limited success in measuring mass concentration [30].”

Line 164: Can authors explain the “atmospheric” concentration channel and their choice to use the “factory” option? What correction factor is applied to the atmospheric condition channels and why was this not used, given the authors’ stated motivation to inform use of these sensors in outdoor air quality measurement scenarios. How do the results for the factory channel apply to the other channel’s measured output? Given both data sets appear, this would be a valuable analysis to provide in supplemental information.

**Reply**: Thanks. According to the sensor manufacturer manual, the factory concentration channel reported mass concentration with a correction factor of 1 applied to the data, which means the number reported for this channel can be viewed as the “raw” data. However, for the atmospheric concentration channel, the manufacturer does not explicitly describe the correction factor and the algorithm applied to the data. The goal of this work is to calibrate the raw readings from the sensor. Therefore, we chose the factory channel as our primary interest for calibration. This is mentioned in the original text.

**pp 9, line 162 - 164 in the revised text:**

“…The mass concentrations are reported for two settings: "factory" and "atmospheric" conditions. The factory condition applies a correction factor (CF) of unity to the concentration measured, whereas the "atmospheric" condition is designed for use in the ambient environment.”

Line 188: Add NaCl to the list.

**Reply**: The text has been revised accordingly.

**pp 10, line 187 - 189 in the revised text:**

“We tested four polydisperse aerosols: Arizona Test Dust (ATD) (Powder Technology Incorporated, Arden Hills, MN), polydisperse W210, and W410 ceramic particles (3M™, St. Paul, MN), and sodium chloride (NaCl) particles.”

Line 314: What mass density was used for the APS mass concentration estimates? The “assumed” density on Table 1? Is the density used in Table 4’s calibration model also the values in Table 1? Last, this sentence only specifies PM2.5 but other mass fractions are also analyzed.

**Reply**: The assumed density in Table 1 for each testing particle was used for the APS mass concentration estimates. The results in Table 4 are also based on the assumed density in Table 1. We have clarified this in the revised text. We have also updated Table 2 with the reference APS bins for calibrating PMS mass concentration.

**pp 18, line 314 - 319 in the revised text:**

“…Based on the same rationale, for mass concentrations, the corresponding APS mass concentration measurement was chosen as the reference for calibrating the PMS mass concentration index. For example, APS PM_2.5_ mass concentration was chosen as the reference (independent variable) for calibrating the PMS PM_2.5_ mass concentration (dependent variable). The assumed density for each testing aerosol in Table 1 was used to obtain the APS mass concentration measurement for calibration.”

**Table 4 in the revised text:**

**“Table 4.** Summary of the calibration model for mass concentration ^a^, R^2^, Bayesian information criterion (BIC), and the normalized mean absolute error (NMAE) of the calibration model.

| **Indices** | **Equation** | **Regression ^b^** | **R^2^** | **BIC** | **NMAE** |
| --- | --- | --- | --- | --- | --- |
| ***Full concentration range (APS total number concentration between 0 – 1000 #/ cm^3^) (n = 4,134)*** | | | | | |
| PM_1_ | Linear | y = 1.06 x | 0.96 | 25852 | 3.11% |
|  | Polynomial | y = 0.76 x + 0.007 x^2^ | 0.97 | 24480 | 2.41% |
|  | Linear + CRI + density | y = 1.13 x + 13.88 CRI - 10.13 density | 0.97 | 24181 | 2.84% |
|  | Polynomial + CRI + density | y = 0.83 x + 0.01x^2^ + 14.44 CRI - 9.58 density | 0.98 | 23432 | 2.33% |
| PM_2.5_ | Linear | y = 2.29 x | 0.94 | 42435 | 4.53% |
|  | Polynomial | y = 1.55 x + 0.006 x^2^ | 0.96 | 41341 | 3.41% |
|  | Linear + CRI + RH | y = 2.51 x - 23.27 CRI + 0.36 RH | 0.95 | 41565 | 4.07% |
|  | Polynomial + CRI + RH | y = 1.80 x + 0.004 x^2^ - 15.55 CRI + 0.42 RH | 0.96 | 41152 | 3.44% |
| PM_10_ | Linear | y = 1.53 x | 0.85 | 49963 | 3.56% |
|  | Polynomial | y = 0.72 x - 0.003 x^2^ | 0.88 | 48959 | 2.61% |
|  | Linear + CRI + RH | y = 1.69 x - 39.14 CRI + 0.56 RH | 0.87 | 49544 | 3.31% |
|  | Polynomial + CRI + RH | y = 0.73 x + 0.003 x^2^ - 17.94 CRI + 0.75 RH | 0.88 | 48931 | 2.61% |
| ***Lower concentration range (APS total number concentration < 100 #/ cm^3^) (n = 1,838)*** | | | | | |
| PM_1_ | Linear | y = 0.72 x | 0.90 | 6211 | 10.10% |
|  | Polynomial | y = 0.91 x - 0.02 x^2asn^ | 0.91 | 6053 | 9.23% |
|  | Linear + CRI + density | y = 0.57 x + 4.80 CRI - 2.68 density | 0.93 | 5746 | 8.16% |
|  | Polynomial + CRI + density | y = 0.80 x - 0.02 x^2^ + 4.93 CRI - 2.95 density | 0.93 | 5694 | 8.08% |
| PM_2.5_ | Linear | y = 1.10 x | 0.91 | 11170 | 9.14% |
|  | Polynomial | y = 1.34 x - 0.01 x^2^ | 0.91 | 11087 | 8.80% |
|  | Linear + CRI + RH | y = 0.97 x -1.87 CRI + 0.16 RH | 0.92 | 10890 | 8.01% |
|  | Polynomial + CRI + RH | y = 1.14 x - 0.006 x^2^ - 2.43 CRI + 0.17 RH | 0.92 | 10885 | 7.97% |
| PM_10_ | Linear | y = 0.63 x | 0.89 | 11878 | 9.30% |
|  | Polynomial | y = 0.86 x - 0.01 x^2^ | 0.90 | 11686 | 8.53% |
|  | Linear + CRI + RH | y = 0.54 x - 2.15 CRI + 0.20 RH | 0.91 | 11627 | 8.01% |
|  | Polynomial + CRI + RH | y = 0.78 x - 0.004 x^2^ - 3.57 CRI + 0.21 RH | 0.91 | 11461 | 7.75% |

^a^ The APS mass concentration measurement was obtained with the assumed density for testing aerosols in Table 1.

^b^ y: APS measurement; x: PMS measurement.

Definition of abbreviations: n = number of datapoints; CRI = complex index of refraction; RH = relative humidity; BIC = Bayesian information criteria; NMAE = normalized mean absolute error.”

**Table 2 in the revised text:**

**“Table 2.** The PMS manufacturer's specified size bins and mass indices with the corresponding reference APS size bins for calibration.

| **PMS indices** | **Reference APS indices** |
| --- | --- |
| ***Number concentration*** |  |
| > 0.3 µm | counts aggregated from all size bins (< 0.523 µm and 0.542 – 19.81 µm) |
| > 0.5 µm | counts aggregated from size bins 0.542 – 19.81 µm |
| > 1 µm | counts aggregated from size bins 1.037 – 19.81 µm |
| > 2.5 µm | counts aggregated from size bins 2.642 – 19.81 µm |
| > 5 µm | counts aggregated from size bins 5.048 – 19.81 µm |
| > 10 µm | counts aggregated from size bins 10.37 – 19.81 µm |
| ***Mass concentration*** |  |
| PM_1_ | mass aggregated from size bin < 0.523 µm – 0.965 µm |
| PM_2.5_ | mass aggregated from size bin < 0.523 µm – 2.458 µm |
| PM_10_ | mass aggregated from size bin < 0.523 µm – 9.647 µm |

”

Line 319: Recommend incorporating into the abstract / conclusions the significant model performance decrease for larger size particles. As noted earlier, recommend PM2.5-10 mass concentration be added as an analysis which will confirm if that size fraction is poorly measured by the sensor.

**Reply**: Thank you. We have rewritten the conclusions accordingly.

**pp 23 - 24, line 391 - 408 in the revised text:**

“In this study, we evaluated the PMS sensors and reported the calibration algorithm for both number concentration and mass concentration. We found that the number concentrations can be corrected using a simple linear model, and mass concentrations can be better corrected using a polynomial model, although the BIC statistical considerations indicated models that including particle properties and environmental conditions are technically superior. When restricting the fit to the lower concentration, the accuracy of the model decreases for both number and mass concentration, and the larger size bins tended to have higher errors. In addition, environmental conditions related to real-world scenarios need to be considered when applying these models. We used particles in a relatively narrow range of CRIs, morphologies, and densities, and our RH was restricted within 20 - 80%. If the particle properties and environmental conditions of interest are significantly different from tested scenarios, one may need to consider these effects. Despite these limitations, this study has major implications, especially for using these sensors in high concentration environments, including indoor air quality monitoring, occupational/industrial exposure assessments, wildfire smoke, or near-source monitoring scenarios. Since the test aerosols used in this study are applicable for several occupational health scenarios, a better exposure assessment could be achieved. In the application of near-source and indoor air quality monitoring where field calibration might be challenging, these low-cost sensors along with the calibration algorithms developed within a higher concentration range applied could serve as a substitute for the traditional gravimetric instruments to provide accurate air quality data.”

Figure 6: The PM1 results are barely viewable, PM2.5 partially blocked. Perhaps reorder the parameters for better viewing.

**Reply**: Figure 6 has been updated for better viewing of the PM_1_ results.

**Revised Figure 6:**

**Fig 6.** A comparison of the pre-calibrated and post-calibrated number concentration by full and lower concentration range. The blue line represents the 1:1 relationship between the PMS and APS concentration.
