# Supplementary material for: Assessing the value of complex refractive index and particle density for calibration of low-cost particle matter sensor for size-resolved particle count and PM2.5 measurements
Source: PLoS One. 2021 Nov 11;16(11):e0259745. doi: 10.1371/journal.pone.0259745 (PMC8584671; doi:10.1371/journal.pone.0259745)
Supplement: Attachment — Submitted filename: Response to Reviewers.docx [file pone.0259745.s020.docx]

***PloS One* Peer Review Comments and Responses**

October 13, 2021

We would like to thank the Editor and Reviewers for their thoughtful comments on how we can improve our manuscript. We reproduce each comment and provide responses below. Additional grammar check was done to improve readability of the manuscript.

**Comments from the Editor**

1. Line 32-38: First the results show that the NAME is about 4% without correction; then when adjusting for CRI and density, the NAME is within 5%. The following sentence then suggests that the calibration algorithms developed in this study...

What this tells us is that the adjusting for CRI and density does not help with the mass measurements by PMS, so this is not needed? Is this correct?

**Reply:** Yes. For mass concentration, the model adjusting for CRI and density yields a slightly lower error than the simple linear and polynomial models (See Table 4). This was clarified in the text:

*“**CRI adjustment did not produce a significantly better fit for mass concentration calibration.*”

2. Can you please make the key message of this paper clearer, in particular what calibration algorithms? Does this refer to the "linear model" as mentioned in line 33 or the model adjusted for CRI and density? If later, then what is the point?

**Reply:** The calibration algorithm refers to the linear model for number concentration and the polynomial model for mass concentration. The conclusions that number concentration could be corrected using a simple linear model, and mass concentrations can be better corrected using a polynomial model. The statement in the conclusion addresses this comment.

*“We found that the PMS's number concentrations can be corrected using a simple linear model, and mass concentrations can be better corrected using a polynomial model. Although the BIC indicated models adjusted for particle properties and environmental conditions are statistically superior, those models did not significantly improve NMAE.”*

3. If I understand correctly, the number size distribution measurements by PMS are not good but the mass measurements are reasonable. Is this correct?

**Reply:** Yes. The number size distribution measurements by PMS did not agree well with the reference instrument. Also, in the top-left panel of Figure 5, we see the PMS significantly underestimated the number concentration of the APS. For the mass concentration, although we observed some non-linearity between the relationship of the APS and PMS, the PMS did not seem to significantly underestimate or overestimate the number concentration of the APS (Figure 6, top-left panel). We have added the following comment to the manuscript:

*“The OEM calibration for PM mass concentration showed significantly better agreement with reference instrument than the OEM number density calibration.”*

Line 209: not a full sentence.

**Reply:** We have revised the sentence. Additional grammar check was done to improve readability of the manuscript.
